# Metronomic and single high-dose paclitaxel treatments produce distinct heterogenous chemoresistant cancer cell populations

**DOI:** 10.1038/s41598-023-46055-6

**Published:** 2023-11-06

**Authors:** Carolina Mejia Peña, Thomas A. Skipper, Jeffrey Hsu, Ilexa Schechter, Deepraj Ghosh, Michelle R. Dawson

**Affiliations:** 1 Department of Molecular Biology, Cell Biology, and Biochemistry, Brown University, Providence, RI 02912 USA; 2 Center for Biomedical Engineering, School of Engineering, Brown University, Providence, RI 02912 USA

**Keywords:** Cancer, Cancer metabolism, Cancer therapy, Gynaecological cancer, Tumour heterogeneity

## Abstract

More than 75% of epithelial ovarian cancer (EOC) patients experience disease recurrence after initial treatment, highlighting our incomplete understanding of how chemoresistant populations evolve over the course of EOC progression post chemotherapy treatment. Here, we show how two paclitaxel (PTX) treatment methods- a single high dose and a weekly metronomic dose for four weeks, generate unique chemoresistant populations. Using mechanically relevant alginate microspheres and a combination of transcript profiling and heterogeneity analyses, we found that these PTX-treatment regimens produce distinct and resilient subpopulations that differ in metabolic reprogramming signatures, acquisition of resistance to PTX and anoikis, and the enrichment for cancer stem cells (CSCs) and polyploid giant cancer cells (PGCCs) with the ability to replenish bulk populations. We investigated the longevity of these metabolic reprogramming events using untargeted metabolomics and found that metabolites associated with stemness and therapy-induced senescence were uniquely abundant in populations enriched for CSCs and PGCCs. Predictive network analysis revealed that antioxidative mechanisms were likely to be differentially active dependent on both time and exposure to PTX. Our results illustrate how current standard chemotherapies contribute to the development of chemoresistant EOC subpopulations by either selecting for intrinsically resistant subpopulations or promoting the evolution of resistance mechanisms. Additionally, our work describes the unique phenotypic signatures in each of these distinct resistant subpopulations and thus highlights potential vulnerabilities that can be exploited for more effective treatment.

## Introduction

High grade serous ovarian cancer (HGSOC) has the highest mortality rate among all gynecological malignancies with the majority of patients experiencing resistance to chemotherapies, including the frontline chemotherapeutic, paclitaxel (PTX)^1^. PTX is an anti-mitotic agent that binds to β-tubulin stabilizing microtubules (MTs) and prevents their disassembly, thus inhibiting mitosis and eventually triggering apoptosis. Cells that survive PTX-induced mitotic arrest through mitotic slippage can also develop multipolar spindles, which has been associated with the formation of polyploid giant cancer cells (PGCCs)^2^. By stabilizing MTs, PTX treatment also reduces the concentration of free tubulin in the cytosol which can lead to metabolic stress. Since free tubulin docks on voltage dependent anion channels (VDACs) on the mitochondrial membrane to maintain their gated structure, dysregulation of this docking mechanism results in increased membrane permeability, altered metabolite flux, changes in bioenergetic processes, and increased reactive oxygen species (ROS)^3–5^. As such, PTX-resistant cancer cells can undergo metabolic reprogramming to cope with PTX-induced metabolic stress.

Cancer stem cells (CSCs) and polypoidal giant cancer cells (PGCCs) are two subpopulations of quiescent cancer cells that likely survive chemotherapy and develop chemoresistance mechanisms through alterations in metabolism. An example of such mechanisms is therapy-induced senescence (TIS). TIS may result in the reprogramming of cancer cells into CSCs^6,7^, which have the ability to use multiple bioenergetic pathways to cope with extrinsic stress^8,9^. Furthermore, CSCs can escape their senescence-associated mitotic arrest to replenish the bulk cancer cell population by reverting to a mitotic phenotype^10,11^. Cancer cells can also undergo polyploidization as a response to stress^12^, forming PGCCs which share many features with CSCs including stem cell and senescent properties^12^, but differ in their ability to give rise to new cells through amitotic budding^13,14^. While it is known that CSCs and PGCCs can endure immediate therapeutic stress, the long-term effects of their metabolic plasticity and resistance phenotypes remain poorly understood.

Here, we illustrate PTX-induced metabolic reprogramming events in the initial surviving populations and fully recovered populations of HGSOC cells cultured in mechanically relevant 3D alginate microsphere cultures over a 4-week period. We considered the impact of weekly low-dose (i.e. metronomic) or single high-dose PTX treatment on surviving cell populations. Using single cell heterogeneity analysis, metabolic profiling, and transcriptional analysis, we show that both PTX treatment regimens produced PTX-resistant subpopulations; however, only high-dose PTX treatment enriched for both CSC and PGCC populations that displayed multiple features of TIS. Whereas, metronomic treatment promoted only a subset of the resistance features that were observed in the high-dose PTX-treated populations. Using untargeted metabolomics, we then probed for the extent of metabolic reprogramming in high-dose PTX treated cells and DMSO control cells after the initial treatment and in the recovered population. We demonstrate using Integrated Pathway Analysis that endogenous antioxidant mechanisms used to cope with PTX-induced oxidative stress present shortly after PTX treatment are only partially retained in fully recovered populations. In summary, we describe the metabolic profile elicited by high dose PTX treatment, which supports the induction of metabolic plasticity in PGCC and CSC populations that survive chemotherapy and shows the extent to which these altered metabolic features persist in cells that stem from these early survivors.

## Results

### Altering PTX dose and frequency generates differences in spheroid number and growth morphology

**Figure 1:**
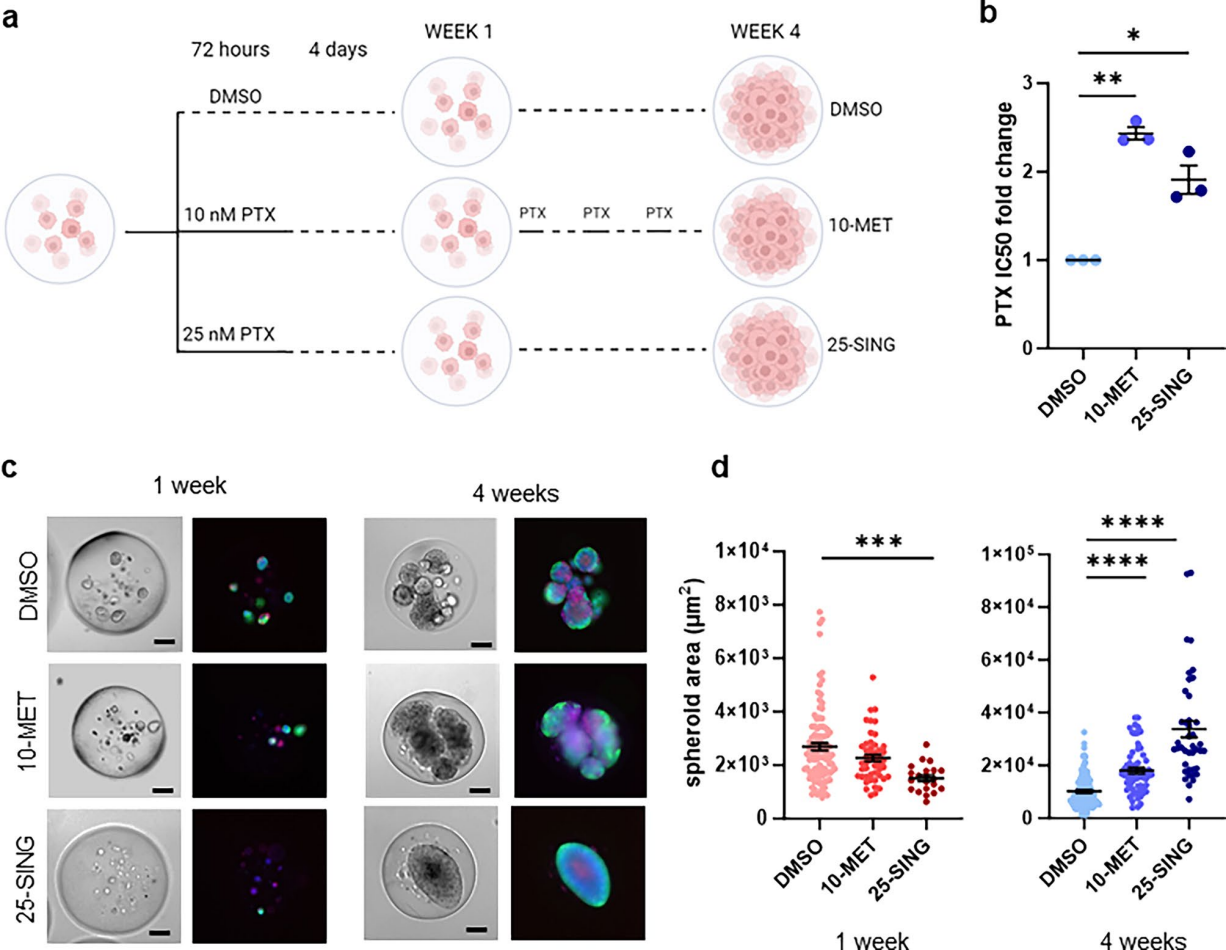
OVCAR8 growth in 3D alginate microspheres illustrates effects of distinct PTX treatment regimens. (**a**) Treatment schedule: control (weekly DMSO administration), metronomic (weekly PTX (10 nM) administration) and acute high-dose (single PTX (25 nM) administration) and sample collection points (1 week and 4 weeks). (**b**) Fold change of PTX IC50 values normalized to respective DMSO samples; (n = 3). (**c**) Representative images of DMSO, 10 nM, and 25 nM treated populations in microspheres at 1 and 4 weeks; left: bright field images, right: live-cell imaging Calcein AM, propidium iodide, Hoechst; scale bar = 100 µm. (**d**) Individual spheroid area calculated by tracing spheroid perimeter in bright field images at 1 week and 4 weeks; (n = 58–208). *p < 0.05, **p < 0.01, ***p < 0.001, ****p < 0.0001.

OVCAR8 cells were encapsulated in alginate-gelatin microspheres by dripping a small volume of cell solution into a stirring calcium chloride solution using a microfluidic pump. Each 0.5 mm diameter microsphere was formed with approximately 100 cells. The microspheres were cultured in normal growth media for 48 h before treating with PTX. We first observed the growth of encapsulated OVCAR8 cells in the presence or absence of PTX over the course of 4 weeks. Encapsulated cells were exposed to weekly DMSO treatments, weekly 10 nM PTX treatments (10-MET), or a single high-dose of 25 nM PTX followed by 3 weeks of recovery in media with DMSO (25-SING) (Fig. 1a). To confirm we had generated PTX-resistant populations, we performed IC50 assays on cells isolated from the microspheres after 4 weeks of treatment and found that both 10-MET and 25-SING populations had an increase in IC50 value when compared to DMSO (Fig. 1b).

At one week the number of viable spheroids per microsphere decreased with increasing PTX concentration (Fig. 1c and Supplemental Fig. 1A). DMSO and 10-MET spheroids were of similar size, while the 25-SING spheroids were ~ 1.7 fold smaller in area compared to control (Fig. 1d). These results suggested 10-MET microspheres had experienced some cell death, but proliferation of the surviving cells appeared to be unaltered given that the spheroid size was similar to DMSO-treated microspheres. In contrast, the 25 nM PTX treatment selected for a small population of OVCAR8 cells with perhaps stunted proliferation given the decrease in both spheroid number and size.

By 4 weeks, we observed clear differences in the growth morphology of DMSO and PTX-treated spheroids (Fig. 1c and Supplemental Fig. 1A). While the median number of viable DMSO-treated spheroids per microsphere remained unchanged, the average had decreased. At 4 weeks, the microspheres were densely populated with cells; thus, the reduced number of spheroids likely resulted from steric interactions or crowding, which may limit the survival of some spheroids or cause them to merge with neighboring spheroids (Supplemental Fig. 1A). Spheroid area increased dramatically within each condition (Fig. 1d). The correlation between spheroid area and PTX concentration can likely be attributed to unrestricted growth conditions due to fewer spheroids per microsphere in PTX-treated microspheres compared to the crowded DMSO-treated microspheres.

As the spheroids were isolated from the microspheres at week 4, we noticed that DMSO and 25-SING spheroids retained their aggregated shape, however, the 10-MET samples quickly dissociated upon release (Supplemental Fig. 1B). One of the key qualities aggressive HGSOC cells must possess is the ability to resist anoikis and survive in suspension in the peritoneal cavity^15^. Observing this, we were curious to see how these populations coped with the stress of suspension culture. At 4 weeks, isolated cells were dissociated into a single cell suspension and immediately cultured in suspension followed by a colony formation assay to assess anoikis resistance. Only the 25-SING population exhibited any difference in colony formation and formed on average nearly 3 times the number of colonies as the DMSO and 10-MET populations suggesting enhanced anoikis resistance (Supplemental Fig. 1C).

Overall, these results illustrate that metronomic and single high-dose administration of PTX impact OVCAR8 spheroid growth and morphology differently. 10 nM PTX treatment results in some cell death but little impact in proliferation. Single high-dose exposure to PTX selects for a small population of OVCAR8 cells at week 1 which develop into large spheroids with accelerated proliferation by week 4. Having characterized overall spheroid morphology, we isolated each population from the microspheres to assess heterogeneity across treatments.

### Surviving high-dose PTX-treated populations are enriched for PGCCs and budding nuclei

**Figure 2:**
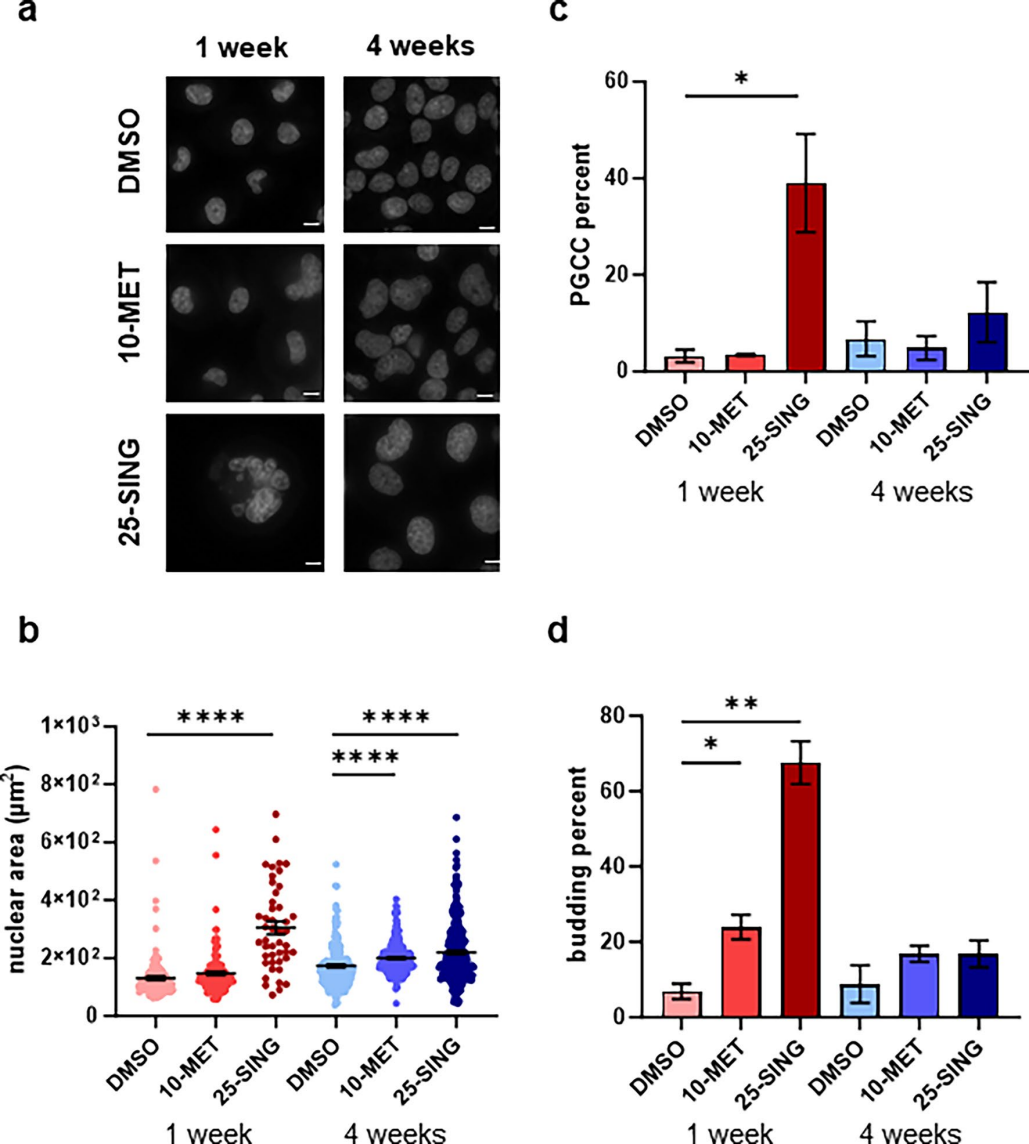
Metronomic and single high-dose regimens generate PTX resistant populations with distinct signatures of nuclear heterogeneity. (**a**) Representative images of nuclei stained with Hoechst at 1 and 4 weeks; scale bar = 10 µm. (**b**) Quantification of nuclear area using nuclear perimeter traces; (n = 122–585). (**c**) Quantification of PGCC fraction; area cutoff was 2.5 × the nuclear average of DMSO 1-week nuclei (330.176 µm). (**d**) Quantification of the fraction of nuclei with evidence of nuclear budding; (n = 122–585). *p < 0.05, **p < 0.01, ***p < 0.001, ****p < 0.0001.

We first quantified the nuclear area of cells plated on coverslips 24 h after isolation from the microspheres at 1 and 4 weeks. We found that the average nuclear area of 25-SING populations increased by more than twofold compared to the control and 10-MET populations at one week (Fig. 2a,b). We previously studied the small yet potent subpopulations of polyploidal giant cancer cells (PGCCs)^16,17^. PGCCs are multinucleated cells often identified in advanced stage disease or post-chemotherapy. Not only are these cells uniquely resistant to PTX, but they can also replenish cancer cell populations via amitotic budding^13,14^. In line with our previous studies, PGCCs were defined as cells with > 2.5 times the average nuclear area^16,17^. The control and 10-MET cells had a small fraction of PGCCs (3%), while PGCCs comprised nearly 40% of the 25-SING populations (Fig. 2c).

By 4 weeks, the distribution of nuclear area was more uniform across the conditions and all conditions experienced an increase in average nuclear area compared to 1 week (Fig. 2b). The 25-SING population still had a significantly larger average nuclear area compared to control populations, however, now by 4 weeks, the 10-MET populations also had larger average nuclear area compared to control (Fig. 2b). The PGCC fractions were also more uniform across all conditions at 4 weeks suggesting that after recovery, the 25-SING population had replenished a bulk population of non-PGCCs (Fig. 2c).

Previous studies have shown that PGCCs can generate daughter cells via amitotic budding^13,14^. The enrichment of PGCCs followed by the recovery of bulk populations at 4 weeks, prompted us to ask whether amitotic budding was present in the initial 25-SING populations. To this end, we quantified the fraction of nuclei with neighboring micronuclei or budding nuclei (Fig. 2d). We found that nearly 70% of nuclei in the initial 25-SING populations had budding nuclei, meanwhile control had around 7%. Interestingly, we found that despite having a similar PGCC fraction to control, the 10 nM treated population had a significantly larger fraction of budding nuclei (Fig. 2d). This result prompted us to ask to what degree the budding and PGCC subpopulations overlapped.

We categorized individual nuclei as either PGCC, budding, both, or neither in order to gauge whether the budding fraction and the PGCC fraction were mutually exclusive (Supplemental Fig. 2A). We found that there was significant overlap in the budding and PGCC subpopulation in the 25-SING group such that nearly all PGCCs were budding (84–95%) and on average half of the cells positive for budding were also PGCCs (38–76%). The subpopulation of budding non-PGCCs increased with PTX treatment in the first week from 2 to 9% in the untreated, 17–26% in the 10-MET, and 18–40% in the 25-SING groups. Nuclear budding can be indicative of amitotic proliferation as well as clearance of damaged DNA through the formation of micronuclei^18–20^. Therefore, in the future, it would be worthwhile to decouple the formation of micronuclei and amitotic proliferation in PGCCs and non-PGCCs to determine whether there is a difference in or characteristic frequency in such populations.

We next asked to what extent are these subpopulations present in recovered populations at 4 weeks. In DMSO-treated groups there was an increase in each of the three subpopulations, however the relative abundance to one another was comparable to that of the 1-week populations (Supplemental Fig. 2A). Both PTX-treated populations were qualitatively more similar to the DMSO control by 4 weeks with the majority of cells being non-budding non-PGCCs.

To further investigate the homogenization of subpopulations at 4 weeks, we quantified nuclear shape factor at 4 weeks (Supplemental Fig. 2B). We found that the average nuclear shape factor was similar between the control and recovered 25-SING populations, however, the nuclear shape factor for the 10-MET populations revealed a bimodal distribution of nuclear shape factors with significantly more cells having elongated nuclei (Supplemental Fig. 2B).

The enrichment of cells potentially undergoing amitotic budding prompted us to further characterize their proliferation status by measuring Ki67 levels via flow cytometry. We found that Ki67 was significantly increased in both PTX-treated populations at 1 week (Supplemental Fig. 3A). Furthermore, despite an overall decrease in the fraction of cells positive for budding, Ki67 was still elevated in PTX-treated populations compared to DMSO control at 4 weeks (Supplemental Fig. 3B). Overall, these results describe unique time dependent and PTX regimen dependent changes to nuclear heterogeneity and proliferative status developed in PTX-resistant populations.

### Single high-dose PTX treatment induces autophagy while metronomic treated populations experience a delayed induction

**Figure 3:**
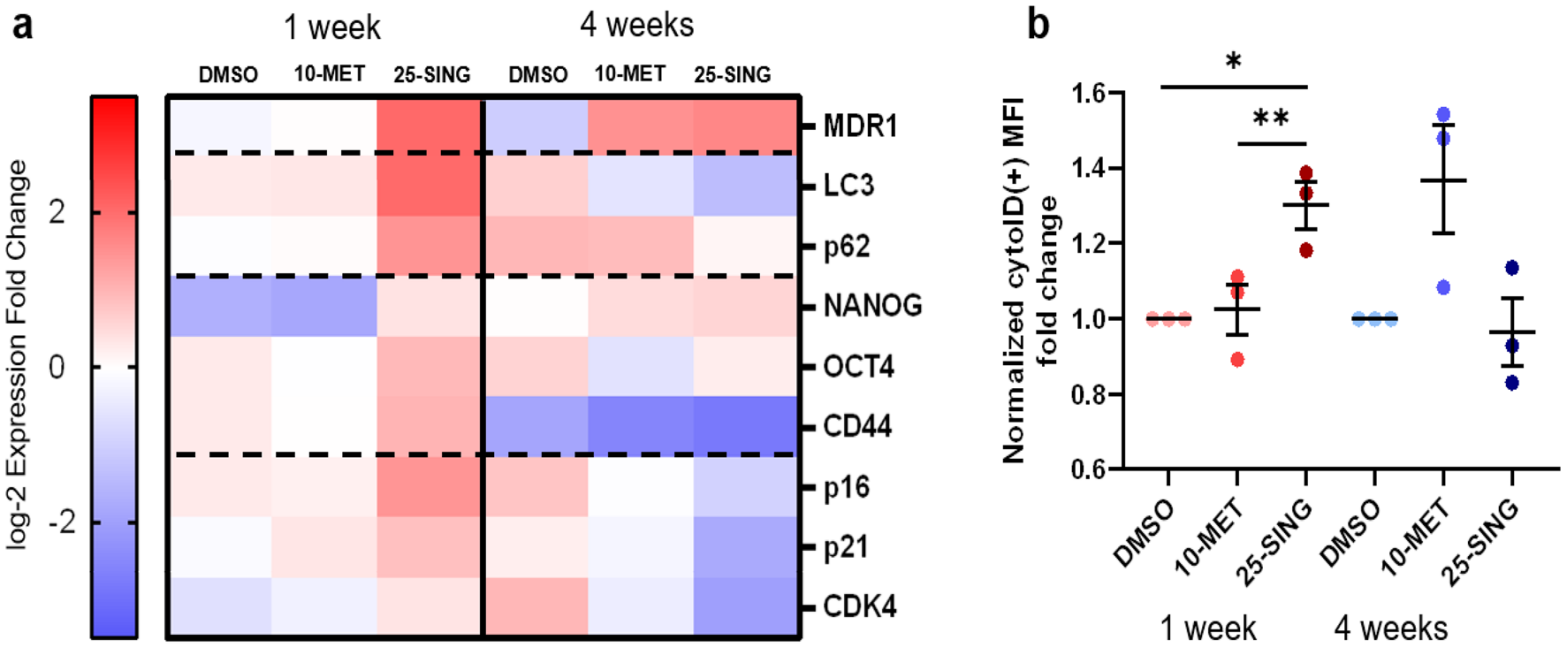
Induction of autophagy are dependent on PTX dose and frequency of administration. (**a**) Heat map of qRT-PCR log2 ΔCq values normalized to 18S; (n = 3). Data normalized using Bio-Rad CFX Maestro Software 1.1 (https://www.bio-rad.com/en-us/product/cfx-maestro-software-for-cfx-real-time-pcr-instruments?ID=OKZP7E15) and heatmap plotted using GraphPad Prism 9.5.1. (**b**) Fold change of median fluorescence intensity (MFI) of cytoID + cells normalized to respective DMSO sample; (n = 3). *p < 0.05, **p < 0.01, ***p < 0.001, ****p < 0.0001.

Based off of previous work from our lab and others, we anticipated that the repeated PTX treatment to the 10-MET group would promote the upregulation of the efflux pump p-glycoprotein (MDR1) which is tightly correlated with PTX resistance^21,22^. Indeed, MDR1 transcript expression was elevated by the fourth week in the 10-MET group, but a single treatment of 10 nM was insufficient to promote MDR1 expression at week 1 (Fig. 3a). In contrast, a single treatment of 25 nM PTX was sufficient to increase expression at week 1 (Fig. 3a). Importantly, these trends were corroborated by an increase in protein expression as well (Supplemental Fig. 4). Furthermore, MDR1 transcriptional expression remained elevated in the 25-SING group after 3 weeks of recovery (Fig. 3a); however, the MDR1 protein expression was not significantly increased at this time point (Supplemental Fig. 4). Despite the upregulation of only MDR1 transcript but not protein, we found that both 10-MET and 25-SING populations had gained PTX resistance by 4 weeks illustrated by an increase in IC50 values (Fig. 1b). It’s possible therefore that the retained upregulation in MDR1 transcript confers PTX resistance by enabling 25-SING cells to more readily increase MDR1 protein expression in response to future PTX exposures. Paired with our findings that the 25-SING populations experience a homogenization in nuclear heterogeneity, the partial reversal in MDR1 activity prompted us to ask if other chemoresistance features followed a similar trajectory.

A master regulator of the cellular response to general stress, including chemotherapy, is autophagy. Autophagy induction is at the crux of several stress-induced signaling cascades including modifications to metabolism, proliferative status, and stem cell features^23–27^. Therefore, we sought to determine whether autophagy was being differentially regulated. At the first week, only the 25-SING populations experienced an upregulation in the expression of autophagy associated markers LC3 and p62, while the 10-MET populations had similar expression levels to the DMSO-treated cells (Fig. 3a). By 4 weeks, the 25-SING populations experienced a downregulation in both markers, while the 10-MET populations had a mixed profile. Interestingly, by 4 weeks, the control populations had the highest relative expression of both autophagy markers; perhaps indicating that by week 4 the DMSO-treated populations were under increased stress from cell crowding in densely populated DMSO-treated microspheres.

To confirm the induction of autophagy, we used flow cytometry to analyze the median fluorescence intensity (MFI) of a live-cell autophagosome dye in the bulk cell population. We found that of cells positive for the dye, 25-SING cells had a significantly higher MFI compared to either DMSO or 10-MET populations (Fig. 3b). By 4 weeks there were no significant differences in MFI between the treatments; though qualitatively, it appears that 10-MET cells might have an increase in signal. Overall, these results demonstrate that the induction of autophagy by PTX is dose dependent at early timepoints, and that perhaps by 4 weeks, repeated exposure to PTX elicits a similar induction in the 10-MET group.

### Temporary induction of autophagy in single high-dose populations is associated with the rise of an ALDH1A1^+^/CD133^+^ cancer stem cell subpopulation

The unique pattern of autophagy induction in the 25-SING population prompted us to ask whether there was a similar temporary shift towards a cancer stem cell (CSC) phenotype. PGCCs have CSC qualities including increased expression of CD44, OCT4, NANOG, and SOX2^28,29^. We found that CSC markers NANOG, OCT4, and CD44 were upregulated only in the 25-SING cells at 1 week (Fig. 3a). NANOG maintained a similar level of expression from 1 to 4 weeks in the 25-SING population, and by week 4, the 10-MET group had comparable NANOG expression. We also quantified the expression of the senescence markers p16, p21, and CDK4 as both PGCCs and CSCs have been reported to undergo therapy-induced senescence (TIS) in response to therapeutic stress^6,7,30^. All three markers were upregulated at week 1 in PTX-treated populations compared to DMSO-treated cells; however, by week 4, all three senescence markers were downregulated in PTX-treated groups compared to control (Fig. 3a).

We next determined the presence ALDH1A1^+^/CD133^+^ subpopulations which have been previously identified as CSCs present in HGSOC^31–34^. Using flow cytometry, we observed the rise in ALDH1A1^+^/CD133^+^ subpopulations by 1 week in the 10-MET and to a greater extent in the 25-SING populations (Supplemental Fig. 5). By 4 weeks, while PTX-treated samples retained a larger fraction of ALDH1A1^+^/CD133^+^ cells compared to DMSO-treated, the populations shrunk compared to the first week. The enrichment for an ALDH1A1^+^/CD133^+^ CSC subpopulation and the upregulation of CSC markers NANOG, OCT4, and CD44 was unique to the 25-SING population. Meanwhile treatment with 10 nM PTX only enriched for the ALDH1A1^+^/CD133^+^ subpopulation without increasing the transcriptional expression of CSC markers.

So far, these results describe the metronomic PTX-treated population as one that develops resistance associated features after repeated exposure to PTX, while the single high-dose PTX treatment selects for highly resilient cells that survive high dose treatment and later give rise to other resistance populations. Importantly, the surviving high-dose PTX population has elevated stem cell properties and the ability to replenish the bulk cancer cell populations to one with new features.

### Metabolomics analysis illustrates similarities between control samples and recovered PTX populations

**Figure 4:**
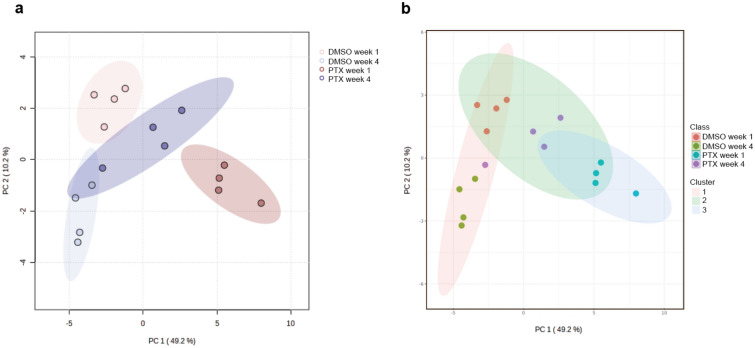
Principle component analysis and K-means clustering illustrates separation between groups and similarities between DMSO control samples and the partial recovery of PTX treated samples by 4 weeks. (**a**) Principle component analysis (PCA) plot and (**b**) K-means clustering sample plot of DMSO and PTX treated samples at 1 and 4 weeks; (n = 4); cluster number = 3. Data was normalized to DMSO 1 week samples, log10 transformed, and scaled using the Pareto method. P-value < 0.05, FDR < 0.05 cut-offs were used.

Thus far we observed the selection for resistance associated properties in the initial 25-SING populations and the subsequent homogenization and replenishment of the bulk population with features similar to those of the control DMSO-treated population. Furthermore, despite the absence of PTX-induced stress, the DMSO-treated population developed characteristics suggesting the accruement of metabolic stress over time (i.e. upregulation of autophagy and senescent markers (Fig. 3a)). This prompted us to further investigate the temporal and treatment contributions to possible metabolic reprogramming events in the DMSO and PTX-treated populations. To this end, we used untargeted metabolomics to characterize the metabolite profiles of week 1 and week 4, DMSO and 25-SING OVCAR8 cells grown in microspheres. Both PCA and PLS-DA demonstrated clear separation between the samples (Fig. 4a and Supplemental Fig. 6). 97 metabolites were detected and 75 were significantly differentially expressed (Supplemental Table 1).

We performed K-cluster analysis and found that the DMSO-treated populations clustered together and that the initial PTX-treated population at 1 week was the most segregated cluster (Fig. 4b). The recovered PTX-treated population at 4 weeks clustered in between the control DMSO cluster and the 1-week PTX cluster. This suggests that the recovered PTX-treated population had retained some features acquired in the first week but by 4 weeks had recovered sufficiently to share features with the control DMSO populations.

### Network analysis predicts sustained detoxification metabolic reprogramming in response to PTX

**Figure 5:**
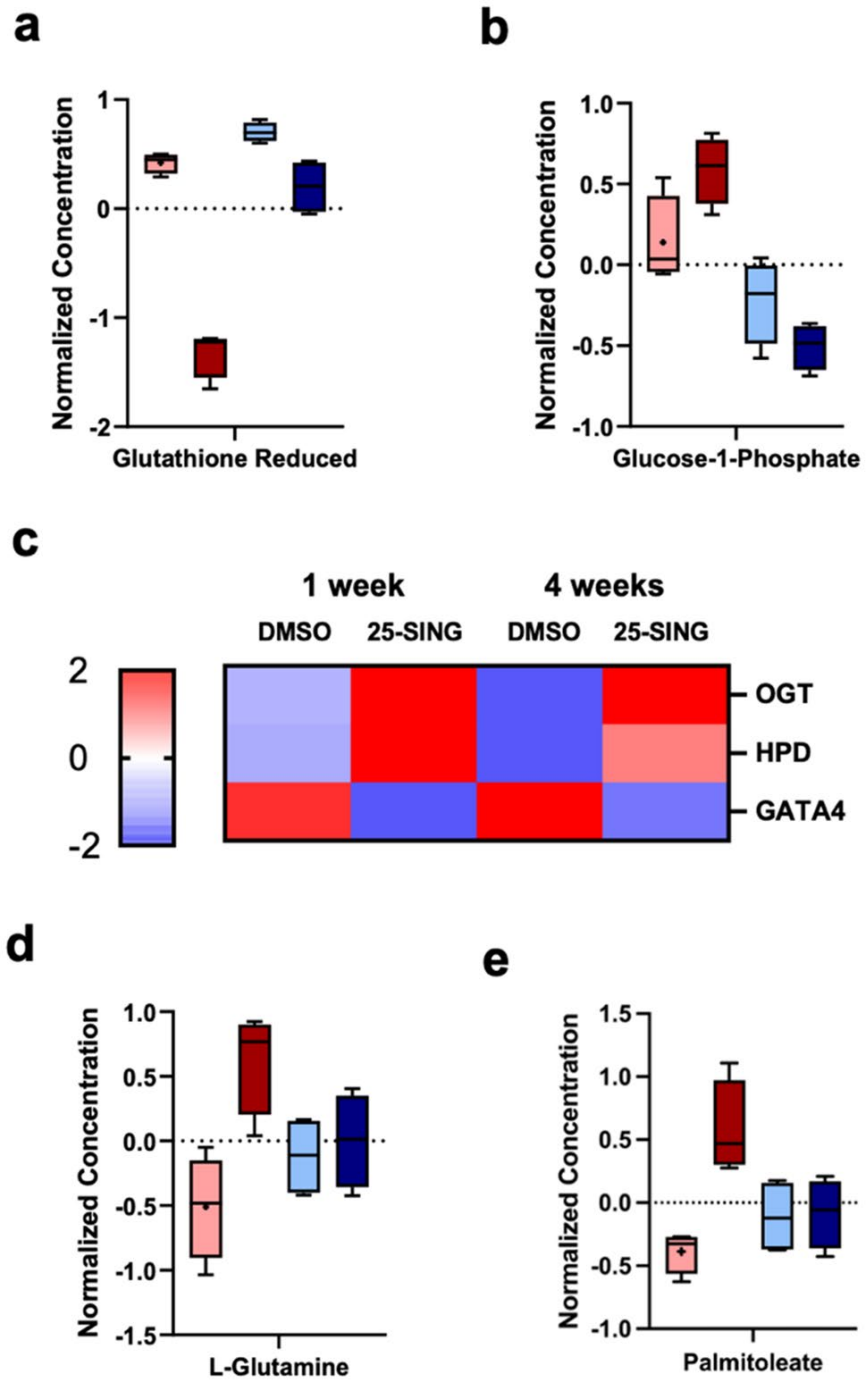
Time and PTX dependent metabolic reprogramming. (**a**) Normalized concentration of glutathione reduced (GSH) and (**b**) glucose-1-phosphate. (**c**) Heatmap of predicted upstream regulators O-GlcNAc transferase (OGT), 4-Hydroxyphenylpyruvate dioxygenase (HPD), and GATA4 with significant activation z-scores. Significant activation z-scores (|2| >) are depicted as |2| for simplicity. Complete list with numerical values are reported in Supplemental Table 2. Data was analyzed using QIAGEN IPA (QIAGEN Inc., https://digitalinsights.qiagen.com/IPA) and plotted using GraphPad Prism 9.5.1. (**d**) Normalized concentrations of l-glutamine and (**e**) palmitoleate.

To begin, we determined whether there was a metabolic profile that was unique to the PTX week 1 group. We found that the antioxidant glutathione reduced (GSH) was the only differentially abundant metabolite in the PTX week 1 group (Fig. 5a). GSH was depleted in cells following PTX treatment but recovered by the fourth week likely reflecting a temporary response to oxidative stressed caused by PTX^4^. In addition to GSH, glucose-1-phosphate was significantly differentially abundant between the 1 and 4-week PTX-treated groups (Fig. 5b). Glucose-1-phosphate is a product of glycogenolysis and can be shunted to the pentose phosphate pathway (PPP) for the synthesis of lipids, nucleotides, or amino acids, or to glycolytic pathways^35^. There was a general temporal trend across the conditions such that glucose-1-phosphate decreased with time, though this trend was exacerbated in the PTX-treated populations. Given that glucose-1-phosphate contributes to a number of biosynthetic pathways including the generation of NADPH and thus the formation of GSH and other detoxifying agents^36^, these differences in relative abundance are likely indicative of changes in proliferation and oxidative stress.

To better understand what metabolic processes these changes in GSH and glucose-1-phosphate reflected, we used Integrated Pathway Analysis (IPA) to predict what cellular functions and pathways were likely activated or inhibited. Using interaction network analysis^37^, we generated molecular networks informed by our metabolomics data to identify and predict the activity of proteins regulating, and regulated by, these metabolites. Additionally, the activity of general cellular functions were predicted using these isolated networks.

The top scored interaction network used the metabolite GSH as a key node (Supplemental Figs. 7–10) and the metabolites listed in Supplemental Table 2. This network included regulators of mitochondrial respiration (cytochrome oxidase and mitochondrial complex 1) and oxidative stress (SOD, catalase (CAT), glutathione peroxidase, glutathione S-transferase (GST), and creatine kinase). There are substantial differences in the predicted activity of these regulators across conditions which are highlighted in three of the cellular functions associated with this network—generation of reactive oxygen species, metabolism of reactive oxygen species, and peroxidation of lipid.

In line with the depletion of GSH, both the generation and metabolism of ROS were predicted to be activated shortly after PTX treatment (Supplemental Fig. 8). The enzyme GST, which detoxifies xenobiotics through the conjugation with GSH^38^, was also predicted to be activated which likely reflects the effort of coping with PTX exposure and explains in part the depletion of GSH.

ROS regulators Sod and CAT were predicted to be activated as well while the inhibition of mitochondrial complex 1 and cytochrome c oxidase would suggest that mitochondrial respiration was likely downregulated. After 3 weeks of recovery following an increase in GSH levels, PTX-treated cells were predicted to have decreased levels of ROS generation and metabolism. Yet, CAT and Sod were still predicted to be activated (Supplemental Fig. 10). Thus, both SOD and CAT were predicted to be activated in response to PTX treatment and associated ROS, yet after 3 weeks of recovery and the stabilization of ROS levels, SOD and CAT remain activated. Their predicted persistent activation could explain an additional mechanism of resistance to subsequent exposures to PTX and associated ROS, in addition to the upregulation of MDR1 (Fig. 3). Additionally, SOD2 was recently found to have a novel epigenetic function promoting cancer cell plasticity where upon acetylation it was converted to a histone demethylase, then localized to the nucleus where it regulated the expression of Nanog and Oct-4^39^. We found Nanog and Oct-4 were upregulated in both week 1 and 4 PTX-treated populations so it’s possible that in addition to serving as an antioxidant upon PTX treatment, Sod may function as a histone demethylase after a period of recovery.

Unlike the PTX-treated cells, control DMSO populations were predicted to have increased mitochondrial respiration, likely due to increased rates of proliferation (Supplemental Fig. 7). While there was some predicted ROS generation, DMSO populations did not have any predicted activation of antioxidant machinery at week 1. Additionally, DMSO populations were unique in that lipid peroxidation was predicted to be highly activated. By 4 weeks, however, glutathione peroxidase was predicted to be activated, and ROS generation and metabolism had stabilized (Supplemental Fig. 9). Therefore, it is likely that as cell number and metabolic demand increased, the DMSO population responded to accruing ROS, and perhaps predominantly from lipid peroxidation, by activating glutathione peroxidase^40^.

Of the nodes generated in this network, creatine kinase was the only enzyme whose predicted activity was dependent on time rather than treatment. By week 4, both DMSO and PTX-treated cells were predicted to have an increase in creatine kinase activity. Creatine kinase plays an important antioxidant role in the formation of ROS by recycling ADP^41^; therefore, the activation of creatine kinase could reflect an antioxidant mechanism that is not relied upon in response to PTX-induced oxidative stress, but rather contributes to detoxification after other mechanisms are employed.

Overall, these predicted activity trends suggest that while both control and PTX-treated cells generate ROS, the contributions of each endogenous antioxidant may differ. Furthermore, despite having reduced ROS levels after a 3-week recovery, its predicted that PTX-treated cells retain elevated activity of CAT and SOD. This raises the possibility that the chemoresistance seen at week 4 could be due in part to enhanced or more rapid detoxification of ROS thus highlighting a critical and sustained metabolic reprogramming event upon first exposure to PTX.

### Potential upstream regulators of metabolic reprogramming and plasticity

Following our network analysis, we turned to the upstream regulator function of IPA to explore whether any predicted candidate genes followed an expression pattern that would explain the priming and sustained changes of detoxifying mechanisms. Only three genes received significant activation z-scores and each had an expression pattern that matched the trends seen in the network analysis (Fig. 5c, Supplemental Table 3).

The first was O-GlcNAc transferase (OGT)—an enzyme in the hexosamine biosynthetic pathway (HBP), which adds the GlcNAc moiety to target proteins and serves as a nutrient sensor through its donor substrate UDP-GlcNAc^42–44^. Because UDP-GlcNAc synthesis requires glucose and glutamine, altering levels of either results in dramatic changes to OGT activity^43^ such that an increase in OGT activity can ultimately stabilize HIF-1α, increase glucose uptake, and promote cell self-renewal^42,43^. Because OGT was predicted to be activated in the PTX-treated populations, we checked to see if there were substantial differences in glutamine concentrations and indeed, the first week PTX-treated cells were uniquely abundant in glutamine (Fig. 5D). Enzymes which participate in the PPP are also regulated by O-GlcNAcylation therefore the unique abundance of glucose-1-phosphate in week 1 PTX-treated cells could reflect an increase in the flux through the PPP pathway. Related, the second candidate, 4-hydroxyphenylpyruvate dioxygenase (HPD), has also been shown to increase PPP flux and was predicted to be activated in the PTX-treated populations. HPD regulates expression of glucose-6-phosphate dehydrogenase, which is a key enzyme that generates NADPH, and thus contributes to the relief of oxidative stress^45^. Lastly, the transcription factor GATA4 was predicted to be inhibited in the PTX-treated populations. GATA4 promotes differentiation in stem cells^46^, yet has been characterized as a tumor suppressor for inhibiting cancer cell proliferation, tumor formation, and inducing senescence^47,48^. Week 1 PTX-treated cells were enriched for PGCCs which had both senescent and stem cell features, therefore it’s difficult to determine how GATA4 activity or inhibition would affect the overall plasticity or proliferative status without further investigation. However, given the rise of a CSCs subpopulation shortly after PTX treatment (Supplemental Fig. 5), we explored whether there was evidence of stemness in the metabolomics data. We found that the unsaturated fatty acid palmitoleate followed a similar trend to that of glutamine such that it was uniquely abundant at 1 week in the PTX-treated cells (Fig. 5e). Unsaturated fatty acids have been repeatedly identified as critical in the maintenance of CSCs^31,49^ thus supporting the notion that week 1 PTX-treated cells were enriched for CSCs.

Altogether, OGT, HBP, and GATA4 represent candidate gatekeepers of the predicted sustained metabolic reprogramming in oxidative stress regulation observed in the PTX-treated populations.

## Discussion

Resistance to the frontline chemotherapeutic PTX persists as a major contributing factor to the high mortality rates in HGSOC^50^. Despite the wealth of literature describing the molecular mechanisms employed by cancer cells to eliminate or cope with PTX, our understanding of how resistant subpopulations are generated and sustained from a heterogeneous cancer population is incomplete. Here, we describe two modes of PTX resistance from a naïve HGSOC population and illustrate the shared and unique properties of the resulting populations. A metronomic treated population (10-MET) developed resistance whereby the features gained over a 4-week period were consistent with the mode of acquired resistance. On the other hand, single high-dose PTX treatment (25-SING) selected for an intrinsically resilient subpopulation of cells which was able to replenish the bulk population and confer resistance associated features to the resulting cancer cells.

Exposure to PTX compromises nuclear structure and integrity by stunting cytokinesis, resulting in endoreplication and mitotic slippage, which ultimately impacts nuclear envelope organization and inhibits nucleocytoplasmic transport^51,52^. It is therefore unsurprising that PTX treated cells exhibit increased heterogeneity in nuclear structure. However, here we show that PTX-induced changes to nuclear heterogeneity are not uniform across PTX treatment regimens and that the resulting populations differ in nuclear profiles with implications in how resistance mechanisms are established and conferred.

The enrichment for PGCCs and a nuclear budding population observed in the 25-SING population provides a mechanism for the transfer of resilient features to the progeny of the initial PTX-treated populations (Fig. 2). Studies have consistently demonstrated that PGCCs exhibit CSC-like and senescent phenotypes^12^ and have the ability to undergo amitotic division^13,14^. Critically, single PGCCs have been shown to generate entire spheroids with enriched CSC features (CD44 and CD133 expression) and enhanced chemoresistance^14^. Our results show that even naïve cancer populations have small PGCC subpopulations which are then enriched for by PTX treatment. In line with emerging canonical PGCC features, the surviving population experienced an increase in both senescence and CSC markers. We also observed the enrichment for budding nuclei and elevated Ki-67 levels, suggesting that the surviving cells were undergoing amitotic division to form mitotic cancer cells. By 4 weeks, Ki-67 levels remained elevated however the PGCC and budding fractions had been diminished to levels similar to the naïve populations suggesting that the recovered populations are proliferating via normal mitosis (Supplemental Fig. 3). Overall, our results give credence to the critical role PGCCs play in replenishing a bulk population as well as conferring chemoresistant traits to their progeny.

The 10-MET population experienced a slight enrichment for budding nuclei upon the first exposure to PTX yet by the fourth week, we observed a non-uniform distribution of nuclear shape factors and a novel subpopulation of OVCAR8 cells with elongated nuclei (Supplemental Fig. 2). Elongated nuclei are characteristic of enhanced motility as well as the epithelial-mesenchymal transition therefore metronomic PTX treatment may be unique in generating an invasive subpopulation unlike the 25-SING population^53,54^. It is possible that long-term PTX exposure elicits gradual cytoskeletal adaptations in addition to PTX resistance. It would be interesting to determine the contributions of this subpopulation to overall PTX resistance.

We were surprised to find a subpopulation of non-PGCCs with budding nuclei across conditions. It is unclear whether non-PGCCs with budding nuclei are diploid cells releasing micronuclei to eliminate DNA damage or non-adherent mitotic cells releasing daughter cells. Additionally, this budding population may represent the last step by which PGCCs undergo depolyploidization to form non-PGCCs. Amitotic division is not exclusive to PGCCs, however further investigation is required to unravel the source and contributions of this subpopulation to tumor growth.

At 1 week, 25-SING cells exhibited a suite of changes including an upregulation of autophagy and senescence associated markers (Fig. 3), and the enrichment of both CSC (Supplemental Fig. 5) and PGCC subpopulations (Fig. 2). Collectively, these features are indicative of therapy induced senescence (TIS). TIS describes a state where therapeutic intervention (e.g., radiation, chemotherapy) drives cancer cells into a dormant state while establishing molecular and metabolic signatures that confer adaptive and resilient properties^11,55^.

Glutamine was uniquely abundant at 1 week in 25-SING cells (Fig. 5). Though a non-essential amino acid, glutamine can serve as an alternative carbon source by feeding the TCA cycle as a anaplerosis metabolite in an environment depleted of nutrients (i.e., glucose)^56,57^. Importantly, glutamine is also a determinant of the induction of TIS in cancer cells such that increased glutamine anaplerosis was found to induce senescence, while the inhibition of mitochondrial glutamine metabolism restricted the induction of senescence^58^. We demonstrated that the initial PTX-treated samples had likely undergone TIS, but by 4 weeks, had replenished the bulk population to one with no senescent features. Glutamine has also been shown to be critical for the escape of TIS in cancer cells. Pacifico et al. found that glutamine depletion inhibits their ability to escape from TIS and furthermore that the glutamine transporter SLC1A5 is overexpressed in cancer cells that successfully escape TIS^59^. Therefore, the temporary increase in abundance of glutamine in the initial PTX-treated populations supports the notion that this population is undergoing TIS, while by 4 weeks the recovered PTX-treated population is one that has escaped TIS.

The upstream regulator OGT, which is also regulated by glutamine availability^43^ was predicted to be activated in PTX treated populations (Fig. 5). OGT and O-GlcNAcylation have prominent roles in promoting and sustaining pluripotent cell states through the regulation of other pluripotent factors^42,43,60^. It is therefore unsurprising that regulation of O-GlcNAcylation also has potent effects on senescence; however, there is no consensus on whether OGT promotes or inhibits cellular senescence^61,62^. Given that the 25-SING population exhibited both stem cell-like and senescent features, the predicted OGT activation in the week 1 could reflect the presence of both. After a long period of recovery, OGT was still predicted to be activated despite a decrease in glutamine concentration (Fig. 5), CSCs subpopulation (Supplemental Fig. 5), and PGCCs (Fig. 2) thus it is possible that OGT is playing a role in maintaining a reprogrammed cell state.

The features best correlated with chemoresistance were the elevated expression of the efflux pump MDR1, and the pluripotency factor, NANOG. Their paralleled expression pattern is in line with the notion that NANOG promotes MDR1 expression via the activation of STAT3^63^. In addition to modulating MDR1 expression, NANOG has been implicated in resistance mechanisms eliciting non-genomic cellular responses, including changes in metabolism, that ultimately generate a resilient cellular phenotype^64–66^. Importantly, GATA4, a direct repressor of NANOG^67^, was predicted to be inhibited in the 25-SING populations (Fig. 5). NANOG is critical in inducing and maintaining pluripotency in cancer cells^68–70^ thus the paralleled inhibition of GATA4 in week 1 likely reflects the enrichment of CSCs and PGCCs. However, after 3 weeks of recovery, the 25-SING population retained an upregulation of NANOG and predicted inhibition of GATA4 despite the bulk population being comprised of non-PGGCs and non-CSCs (Supplemental Fig. 5 and Fig. 2). These results highlight a likely reprogramming in cell state conferred to a replenished “normal” cancer cell population.

In this study we have illustrated the genesis and described the unique properties of PTX-resistant OVCAR8 populations generated by a metronomic low-dose or single high-dose treatment regimen. By comparing these two commonly interchanged methodologies, we demonstrated that there are substantial differences in nuclear and phenotypic heterogeneity between populations with that acquire resistant features over time, and those replenished by an intrinsically resistant subpopulation. We present evidence that suggests these populations are differentially primed to cope with future insults of chemotherapy or stress associated with metastasis. Furthermore, our metabolomics analysis supports the notion of a TIS profile in the 25-SING populations and illustrated the dynamicity of metabolic reprogramming throughout HGSOC progression and treatment. We hope our findings inform the design of future studies focused on the evolution of chemoresistance in HGSOC and provide a framework for therapeutic interventions tailored to specific chemoresistant states with unique metabolic vulnerabilities.

## Materials and methods

### Cell culture and PTX treatment

OVCAR8 cells were obtained as a gift from Dr. Alex Brodsky and cultured in RPMI 1640 (Corning, USA) supplemented with 10% Fetal Bovine Serum (FBS) (Atlanta Biologicals, USA) and 1% penicillin streptomycin (Corning, USA). Cells were cultured in standard 2D culture conditions or in 3D culture using alginate or alginate-gelatin microspheres with media changed every 3–4 days, unless otherwise specified. Cells were treated with DMSO (control) or Paclitaxel (PTX) solubilized in DMSO. The single dose 25 nM PTX treatment was administered to microspheres the day after encapsulation, and 10 nM PTX treatment was administered at the same time and weekly after that for up to 4 weeks.

### Alginate microsphere production

The alginate-gelatin solutions (1.0% w/v alginate, 0.5% w/v gelatin) (Protanal LF 200 FTS; FMC and Fisher Science Education) were made in sterile-filtered PBS and allowed to shake in 37 °C for 24 h prior to microsphere production. Alginate-gelatin solutions were then sterile-filtered using 0.22 µm filter and combined with OVCAR-8 cell solutions at equal volumes to form 1% alginate with 0.5% gelatin cell solutions with 1.5 × 10^6^ cells per ml. Approximately 100 OVCAR-8 cells were encapsulated in each microsphere. These solutions were then extruded from 35-gauge needle into spinning 100 mM CaCl_2_ using a syringe pump with 1.5 ml per minute continuous flow rate. After 10 min, the resulting microspheres were placed into fresh RPMI 1640 media for cell culture.

### Live-cell imaging

Microspheres were dyed with Calcein AM (50 ng/ml), Hoechst 33342 (1 µg/ml), and propidium iodide (1 µg/ml) in RPMI 1640 for 45 min at 37 °C. Media was removed, and microspheres were immobilized in polymerized agarose (1%) topped with fresh media, for stabilization during live-cell imaging. Gels were imaged using an inverted Nikon Eclipse Ti microscope with an environmental chamber set to 37 °C and 5% CO_2_.

### Microsphere chelation and cell isolation

Microspheres were removed from media and washed twice with sterile PBS. Microspheres were then chelated with a sterile filtered solution of EDTA (30 mM), sodium citrate (50 mM), and NaCl (150 mM) in 1 × volume of the microspheres, for 5 min shaking in 37 °C. Chelated samples were then spun down and washed twice with sterile PBS. Cells were then plated or collected for subsequent analyses.

### Flow cytometry

Isolated cells were fixed with paraformaldehyde (4%) for 15 min at 25 °C. Fixed cells were then washed with PBS and then permeabilized with methanol (90%) in − 20 °C overnight. Permeabilized cells were then incubated with primary antibody in BSA (0.5%) for 1 h at 25 °C. Cells were then washed with PBS and then incubated with secondary antibody in BSA (0.5%) for 30 min. Cells were then washed, resuspended in PBS, plated in replicates of 3 and analyzed using an easyCyte HT (Guava instruments) flow cytometer.

### RNA isolation and qRT-PCR

RNA was isolated using RiboZol RNA isolation reagent (VWR) according to the manufacturer’s recommendations. RNA concentration and quality were verified by spectrophotometry (Nanodrop 1000; Thermo Fisher Scientific). gDNA removal and cDNA synthesis were performed using the iScript gDNA Clear cDNA Synthesis Kit (Bio-Rad) according to the manufacturer’s protocol. 1 μg of RNA was converted into cDNA for real-time PCR analysis.

### CytoID autophagy analysis

Cells isolated from microspheres were then immediately incubated with the autophagy indicator cytoID (Promega) according to the manufacturer’s recommendations, washed with PBS, and then analyzed using an easyCyte HT (Guava instruments) flow cytometer.

### PTX resistance assay

Cells were plated at a concentration of 1 × 10^4^ cells/ml in 96 well plates and treated with varying concentrations of PTX for 72 h. Following treatment, cells were incubated with the cell viability reagent CellTiter Blue (Promega) according to the manufacturer’s recommendations. Fluorescence was measured using a DTX 880 plate reader (Beckman Coulter). IC50 values were determined by fitting results to a four-parameter sigmoidal curve using GraphPad software.

### Anoikis assay

Cells isolated from microspheres were immediately plated at a concentration of 3 × 10^4^ cells/ml on poly-2-hydroxyethyl methacrylate (20 mg/ml) coated 12-well plates and cultured in suspension for 48 h. Cells were then allowed to adhere for 24 h after which cells were fixed with PFA (4%) and stained with gentian violet (0.5% in methanol). Wells were imaged using brightfield microscopy and well coverage was determined based on area ratio (cell area to well area) using Fiji software.

### Image analysis

Multicellular spheroids (MCS) and cell nuclear area were determined using Fiji software. Brightfield images of microspheres were used to determine the perimeter of each spheroid, only spheroids that were in the plane of focus were traced manually to determine area. Similarly, Hoechst images were used to identify and trace nuclei to determine nuclear area.

### Metabolomics sample collection and prep

OVCAR8 encapsulated cells were isolated from microspheres as previously described. DMSO 1-week, PTX 1-week, DMSO 4 week, PTX 4 week cell pellets of at least 200 mg were collected in n = 4 for a total of 16 samples. Cell pellets were then flash frozen and stored in − 80 °C.

### Metabolomics untargeted panel (LC–MS)

Untargeted metabolomics was performed (Eremid; Discovery Panel) on the 16 samples. Extracts from samples were processed using a methanol-chloroform solvent containing internal standards for data normalization. Two aliquots of extract were dried down for analysis. GC × GC–MS aliquots will be derivatized with methoxyamine in pyridine and MSTFA + 1% TMCS. UPLC-MS/MS aliquots were reconstituted with a methanol–water solution. Extracts were then analyzed by GC × GC–MS and UPLC-MS/MS.

### Metabolomics analysis

Metaboanalyst 5.0 was used to perform PCA and PLSDA to assess sample separation as well as K-means clustering. Data was normalized to DMSO 1-week samples, log10 transformed, and scaled using the Pareto method. p-value < 0.05, FDR < 0.05 cut-offs were used.

Integrated Pathway Analysis (QIAGEN) was used for generated integrated network maps. p-value < 0.05, FDR < 0.05 cut-offs were used.

### Statistical analysis

All experiments were done with at least 3 biological replicates and a minimum of 3 technical replicates, unless otherwise noted. Student’s *t*-tests were used to compare two conditions with p-value of less than 0.05 considered significant (*p < 0.05, **p < 0.01, ***p < 0.001). Data is represented as the mean ± standard error of the mean for at least 3 experiments. qRT-PCR data was analyzed using the CFX Maestro (BioRad) software.

### Supplementary Information

Supplementary Information.

## Data Availability

All data are available in the main text or the supplementary materials.

## References

[1] Lee AH, Mejia Peña C, Dawson MR (2022). Comparing the secretomes of chemorefractory and chemoresistant ovarian cancer cell populations. Cancers.

[2] Zasadil LM (2014). Cytotoxicity of paclitaxel in breast cancer is due to chromosome missegregation on multipolar spindles. Sci. Transl. Med..

[3] Varbiro G, Veres B, Gallyas F, Sumegi B (2001). Direct effect of Taxol on free radical formation and mitochondrial permeability transition. Free Radic. Biol. Med..

[4] Chavez JD, Keller A, Zhou B, Tian R, Bruce JE (2019). Cellular interactome dynamics during paclitaxel treatment. Cell Rep..

[5] Maldonado EN, Patnaik J, Mullins MR, Lemasters JJ (2010). Free tubulin modulates mitochondrial membrane potential in cancer cells. Cancer Res..

[6] Mongiardi MP, Pellegrini M, Pallini R, Levi A, Falchetti ML (2021). Cancer response to therapy-induced senescence: A matter of dose and timing. Cancers (Basel).

[7] Milanovic M (2018). Senescence-associated reprogramming promotes cancer stemness. Nature.

[8] Pastò A (2014). Cancer stem cells from epithelial ovarian cancer patients privilege oxidative phosphorylation, and resist glucose deprivation. Oncotarget.

[9] Anderson AS, Roberts PC, Frisard MI, Hulver MW, Schmelz EM (2014). Ovarian tumor-initiating cells display a flexible metabolism. Exp. Cell Res..

[10] Erenpreisa J, Cragg MS (2013). Three steps to the immortality of cancer cells: Senescence, polyploidy and self-renewal. Cancer Cell Int..

[11] Saleh T, Tyutyunyk-Massey L, Gewirtz DA (2019). Tumor cell escape from therapy-induced senescence as a model of disease recurrence after dormancy. Cancer Res..

[12] Song Y, Zhao Y, Deng Z, Zhao R, Huang Q (2021). Stress-induced polyploid giant cancer cells: Unique way of formation and non-negligible characteristics. Front. Oncol..

[13] White-Gilbertson S (2020). Tamoxifen is a candidate first-in-class inhibitor of acid ceramidase that reduces amitotic division in polyploid giant cancer cells—Unrecognized players in tumorigenesis. Cancer Med..

[14] Zhang S (2014). Generation of cancer stem-like cells through the formation of polyploid giant cancer cells. Oncogene.

[15] Sawyer BT (2020). Targeting fatty acid oxidation to promote anoikis and inhibit ovarian cancer progression. Mol. Cancer Res..

[16] Xuan B, Ghosh D, Cheney EM, Clifton EM, Dawson MR (2018). Dysregulation in actin cytoskeletal organization drives increased stiffness and migratory persistence in polyploidal giant cancer cells. Sci. Rep..

[17] Xuan B, Ghosh D, Jiang J, Shao R, Dawson MR (2020). Vimentin filaments drive migratory persistence in polyploidal cancer cells. Proc. Natl. Acad. Sci. U.S.A..

[18] Li X, Zhong Y, Zhang X, Sood AK, Liu J (2023). Spatiotemporal view of malignant histogenesis and macroevolution via formation of polyploid giant cancer cells. Oncogene.

[19] Kneissig M (2019). Micronuclei-based model system reveals functional consequences of chromothripsis in human cells. eLife.

[20] Jagetia GC, Adiga SK (1997). Correlation between micronuclei induction and cell survival in V79 cells exposed to paclitaxel (Taxol) in conjunction with radiation. Mutat. Res. Fundam. Mol. Mech. Mutag..

[21] Mechetner E (1998). Levels of multidrug resistance (MDR1) P-glycoprotein expression by human breast cancer correlate with in vitro resistance to taxol and doxorubicin. Clin. Cancer Res..

[22] McGrail DJ (2015). Alterations in ovarian cancer cell adhesion drive taxol resistance by increasing microtubule dynamics in a FAK-dependent manner. Sci. Rep..

[23] Kimmelman AC, White E (2017). Autophagy and tumor metabolism. Cell Metab..

[24] Pagotto A (2017). Autophagy inhibition reduces chemoresistance and tumorigenic potential of human ovarian cancer stem cells. Cell Death Dis..

[25] Peng Q (2017). Autophagy maintains the stemness of ovarian cancer stem cells by FOXA2. J. Exp. Clin. Cancer Res..

[26] Wang Q (2018). Autophagy is indispensable for the self-renewal and quiescence of ovarian cancer spheroid cells with stem cell-like properties. Oxid. Med. Cell. Longev..

[27] Towers CG, Wodetzki D, Thorburn A (2019). Autophagy and cancer: Modulation of cell death pathways and cancer cell adaptations. J. Cell Biol..

[28] Niu N, Mercado-Uribe I, Liu J (2017). Dedifferentiation into blastomere-like cancer stem cells via formation of polyploid giant cancer cells. Oncogene.

[29] Gerashchenko BI (2016). Disentangling the aneuploidy and senescence paradoxes: A study of triploid breast cancers non-responsive to neoadjuvant therapy. Histochem. Cell Biol..

[30] Stallaert W (2022). The structure of the human cell cycle. Cell Syst..

[31] Li J (2017). Lipid desaturation is a metabolic marker and therapeutic target of ovarian cancer stem cells. Cell Stem Cell.

[32] Parrales A, Ranjan A, Iwakuma T (2017). Unsaturated fatty acids regulate stemness of ovarian cancer cells through NF-κB. Stem Cell Investig..

[33] Foster R, Buckanovich RJ, Rueda BR (2013). Ovarian cancer stem cells: Working towards the root of stemness. Cancer Lett..

[34] Flesken-Nikitin A, Odai-Afotey AA, Nikitin AY (2014). Role of the stem cell niche in the pathogenesis of epithelial ovarian cancers. Mol. Cell. Oncol..

[35] Zois CE, Harris AL (2016). Glycogen metabolism has a key role in the cancer microenvironment and provides new targets for cancer therapy. J. Mol. Med. (Berl.).

[36] Fernandez-Marcos PJ, Nóbrega-Pereira S (2016). NADPH: New oxygen for the ROS theory of aging. Oncotarget.

[37] Krämer A, Green J, Pollard J, Tugendreich S (2014). Causal analysis approaches in ingenuity pathway analysis. Bioinformatics.

[38] Ferracini AC (2021). GSTP1 and ABCB1 polymorphisms predicting toxicities and clinical management on carboplatin and paclitaxel-based chemotherapy in ovarian cancer. Clin. Transl. Sci..

[39] Coelho DR (2022). Nuclear-localized, iron-bound superoxide dismutase-2 antagonizes epithelial lineage programs to promote stemness of breast cancer cells via a histone demethylase activity. Proc. Natl. Acad. Sci..

[40] Jablonska E (2015). Lipid peroxidation and glutathione peroxidase activity relationship in breast cancer depends on functional polymorphism of GPX1. BMC Cancer.

[41] Meyer LE (2006). Mitochondrial creatine kinase activity prevents reactive oxygen species generation: Antioxidant role of mitochondrial kinase-dependent ADP re-cycling activity. J. Biol. Chem..

[42] Ferrer CM (2014). O-GlcNAcylation regulates cancer metabolism and survival stress signaling via regulation of HIF-1 pathway. Mol. Cell.

[43] Swamy M (2016). Glucose and glutamine fuel protein O-GlcNAcylation to control T cell self-renewal and malignancy. Nat. Immunol..

[44] Mannino MP, Hart GW (2022). The beginner’s guide to O-GlcNAc: From nutrient sensitive pathway regulation to its impact on the immune system. Front. Immunol..

[45] Shan C (2019). 4-hydroxyphenylpyruvate dioxygenase promotes lung cancer growth via pentose phosphate pathway (PPP) flux mediated by LKB1-AMPK/HDAC10/G6PD axis. Cell Death Dis..

[46] Fujikura J (2002). Differentiation of embryonic stem cells is induced by GATA factors. Genes Dev..

[47] Gong Y (2018). GATA4 inhibits cell differentiation and proliferation in pancreatic cancer. PLoS One.

[48] Gao L (2019). Lung cancer deficient in the tumor suppressor GATA4 is sensitive to TGFBR1 inhibition. Nat. Commun..

[49] Zhao G (2022). Ovarian cancer cell fate regulation by the dynamics between saturated and unsaturated fatty acids. Proc. Natl. Acad. Sci..

[50] Maloney SM, Hoover CA, Morejon-Lasso LV, Prosperi JR (2020). Mechanisms of taxane resistance. Cancers (Basel).

[51] Gatti G (2009). MYC prevents apoptosis and enhances endoreduplication induced by paclitaxel. PLoS One.

[52] Theodoropoulos PA (1999). Taxol affects nuclear lamina and pore complex organization and inhibits import of karyophilic proteins into the cell nucleus1. Cancer Res..

[53] Grosser S (2021). Cell and nucleus shape as an indicator of tissue fluidity in carcinoma. Phys. Rev. X.

[54] Leggett SE (2016). Morphological single cell profiling of the epithelial–mesenchymal transition. Integr. Biol. (Camb.).

[55] Prasanna PG (2021). Therapy-induced senescence: Opportunities to improve anticancer therapy. JNCI J. Natl. Cancer Inst..

[56] Yang L (2014). Metabolic shifts toward glutamine regulate tumor growth, invasion and bioenergetics in ovarian cancer. Mol. Syst. Biol..

[57] Le A (2012). Glucose-independent glutamine metabolism via TCA cycling for proliferation and survival in B cells. Cell Metab..

[58] Kim B, Gwak J, Lee EK, Jeong SM (2020). Mitochondrial glutamine metabolism determines senescence induction after chemotherapy. Anticancer Res..

[59] Pacifico F (2021). Glutamine promotes escape from therapy-induced senescence in tumor cells. Aging (Albany NY).

[60] Jang H (2012). O-GlcNAc regulates pluripotency and reprogramming by directly acting on core components of the pluripotency network. Cell Stem Cell.

[61] Lee J-S, Zhang Z (2016). O-linked N-acetylglucosamine transferase (OGT) interacts with the histone chaperone HIRA complex and regulates nucleosome assembly and cellular senescence. Proc. Natl. Acad. Sci..

[62] Efimova EV (2019). O-GlcNAcylation enhances double strand break repair, promotes cancer cell proliferation and prevents therapy-induced senescence in irradiated tumors. Mol. Cancer Res..

[63] Bourguignon LYW, Peyrollier K, Xia W, Gilad E (2008). Hyaluronan-CD44 interaction activates stem cell marker Nanog, Stat-3-mediated MDR1 gene expression, and ankyrin-regulated multidrug efflux in breast and ovarian tumor cells. J. Biol. Chem..

[64] Qin S, Li Y, Cao X, Du J, Huang X (2017). NANOG regulates epithelial–mesenchymal transition and chemoresistance in ovarian cancer. Biosci. Rep..

[65] Chen C-L (2016). NANOG metabolically reprograms tumor-initiating stem-like cells through tumorigenic changes in oxidative phosphorylation and fatty acid metabolism. Cell Metab..

[66] Machida K (2018). NANOG-dependent metabolic reprogramming and symmetric division in tumor-initiating stem-like cells. Adv. Exp. Med. Biol..

[67] Serrano F (2013). Gata4 blocks somatic cell reprogramming by directly repressing Nanog. Stem Cells.

[68] Yin X (2015). Coexpression of gene Oct4 and Nanog initiates stem cell characteristics in hepatocellular carcinoma and promotes epithelial–mesenchymal transition through activation of Stat3/Snail signaling. J. Hematol. Oncol..

[69] Zhang C (2016). Hypoxia induces the breast cancer stem cell phenotype by HIF-dependent and ALKBH5-mediated m6A-demethylation of NANOG mRNA. Proc. Natl. Acad. Sci..

[70] Huang C (2020). ERK1/2-Nanog signaling pathway enhances CD44(+) cancer stem-like cell phenotypes and epithelial-to-mesenchymal transition in head and neck squamous cell carcinomas. Cell Death Dis..

