# Supplementary material for: Metronomic and single high-dose paclitaxel treatments produce distinct heterogenous chemoresistant cancer cell populations
Source: Sci Rep. 2023 Nov 6;13:19232. doi: 10.1038/s41598-023-46055-6 (PMC10628134; doi:10.1038/s41598-023-46055-6)
Supplement: Supplementary file 1 — Supplementary Information. [file 41598_2023_46055_MOESM1_ESM.docx]

**Title**

Metronomic and Single High-dose Paclitaxel Treatments Produce Distinct Heterogenous Chemoresistant Cancer Cell Populations

**Authors**

Carolina Mejia Peña^1^, Thomas A. Skipper^2,3^, Jeffrey Hsu^1,4^, Ilexa Schechter^1^, Deepraj Ghosh^1^, Michelle R. Dawson^1,2*^

**Affiliations**

1. Brown University, Department of Molecular Biology, Cell Biology, and Biochemistry, Providence, RI, 02912, USA.

2. Brown University, School of Engineering, Center for Biomedical Engineering, Providence, RI, 02912, USA.

* Correspondence addressed to michelle_dawson@brown.edu

**Supplementary Figures and Tabl****e**


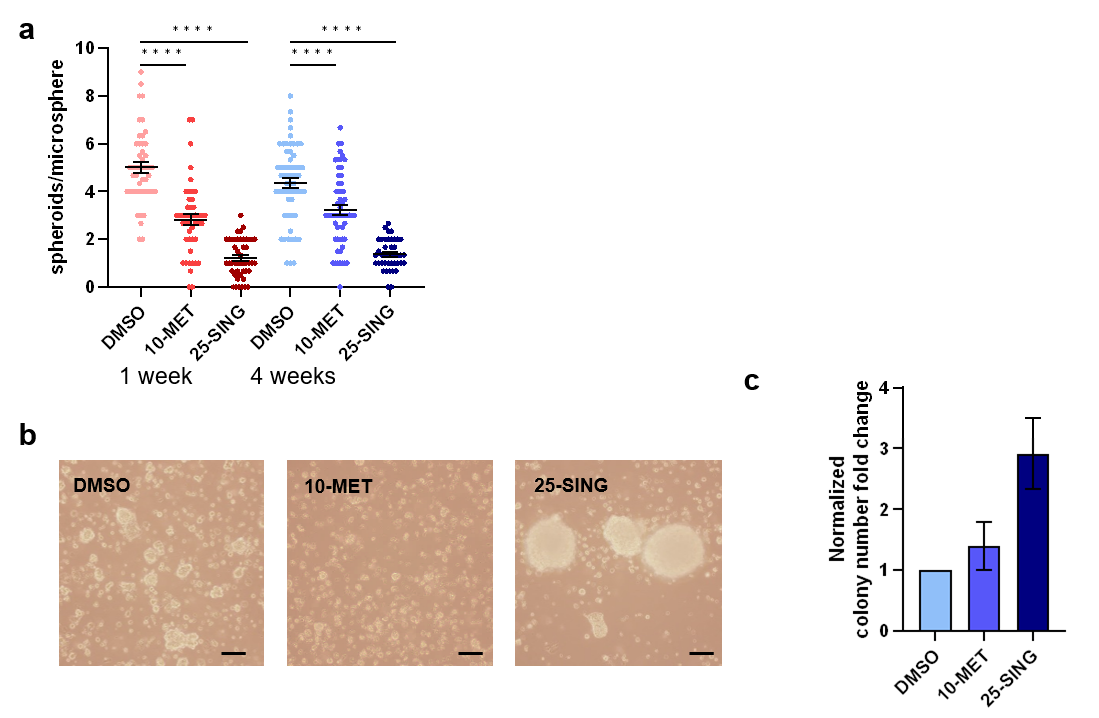


**Supplemental Figure 1. Anoikis resistance is present in 25-SING populations.** (A) Quantification of number of spheroids per microsphere; (n=89-120). (B) Brightfield images of upon release from microspheres at 4 weeks. (C) Anoikis resistance assay quantifying the fold change in colony number after culture in suspension. *p<0.05, **p<0.01, ***p<0.001, ****p<0.0001.


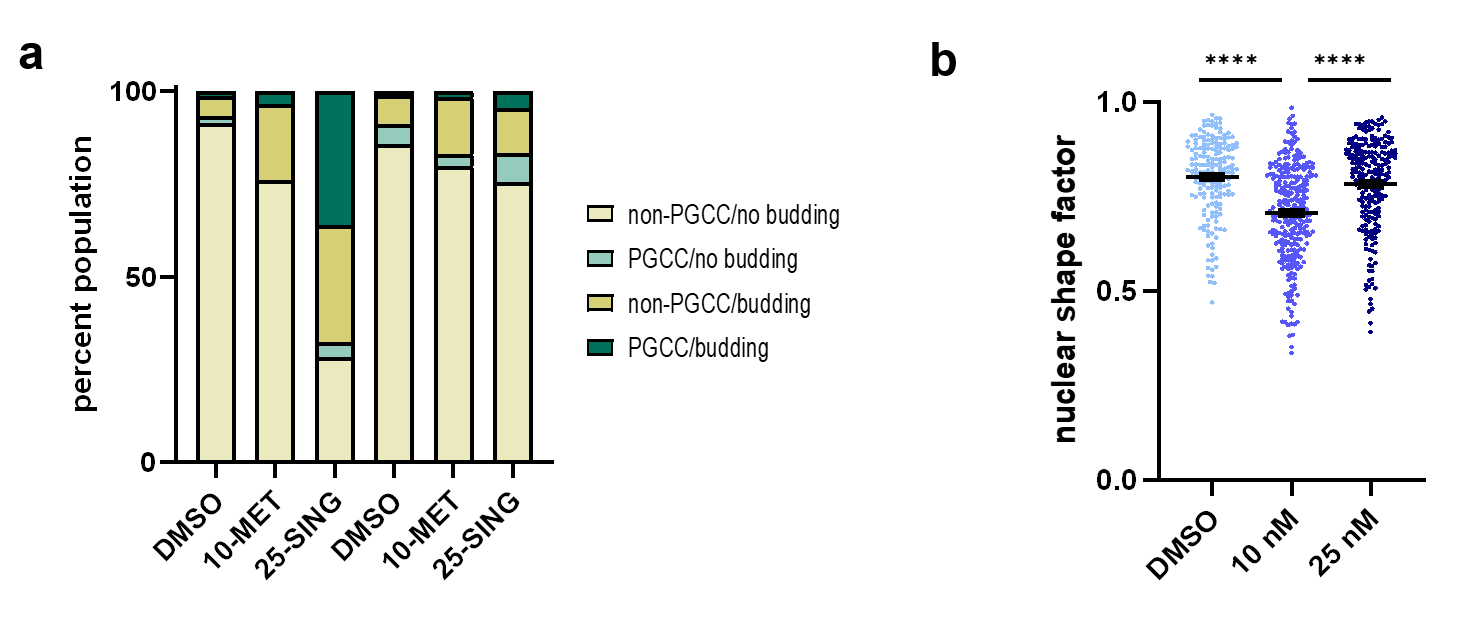


**Supplemental Figure 2. Metronomic and single high-dose regimens generate PTX resistant populations with distinct signatures of nuclear heterogeneity.** (A) Fraction of nuclei that were positive or negative for either or both PGCC and budding; (n=122-585). (B) Quantification of nuclear shape factor using nuclear perimeter traces; (n=184-283). *p<0.05, **p<0.01, ***p<0.001, ****p<0.0001.


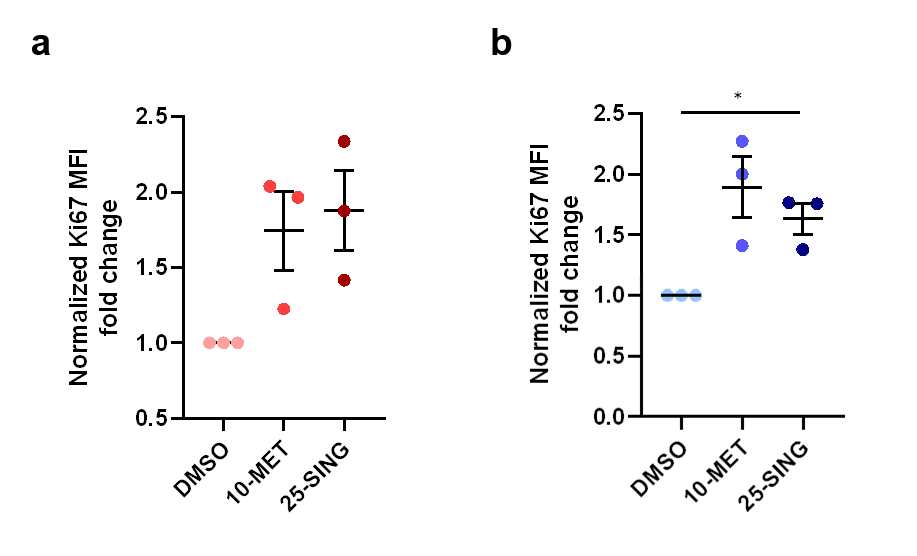


**Supplemental Figure 3.** Fold change of mean fluorescence intensity (MFI) of Ki67+ cells normalized to respective DMSO sample; (n=3) at week 1 (A) and week 4 (B). *p<0.05, **p<0.01, ***p<0.001, ****p<0.0001.


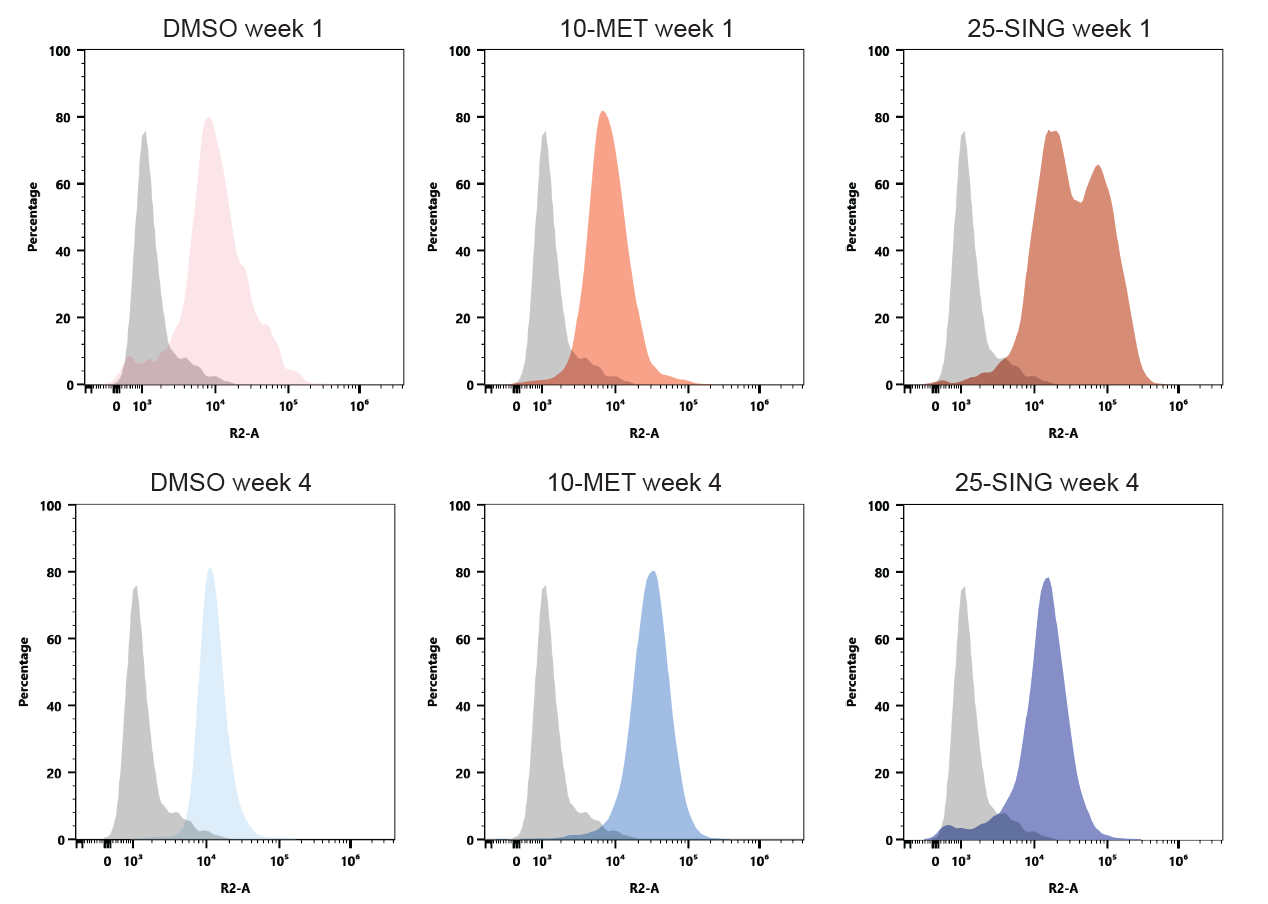


| **Condition** | **MFI** |
| --- | --- |
| DMSO week 1 | 9181 |
| 10-MET week 1 | 7517 |
| 25-SING week 1 | 29335 |
| DMSO week 4 | 11417 |
| 10-MET week 4 | 28967 |
| 25-SING week 4 | 13487 |

**Supplemental Figure 4. MDR1 protein expression follows transcript expression in first week but deviates at fourth.** MDR1 protein expression was assessed via flow cytometry (Cell Signaling MDR1/ABCB1 (E1Y7B); Alexa Fluor 647 Conjugate); Median fluorescence intensity is reported in table. (n=1).


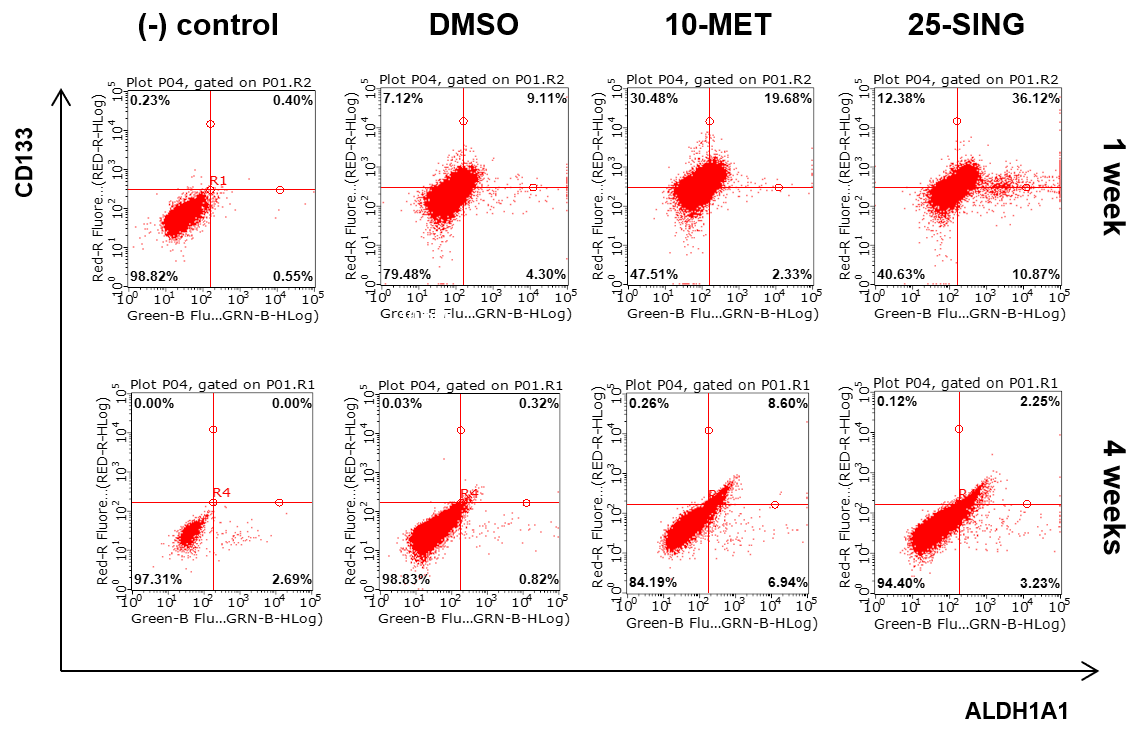


**Supplemental Figure 5. Differential metabolic signatures and stem-cell profiles based on PTX dose and frequency of administration.** (A) Representative flow cytometry plots of cells stained with the cancer stem cell markers ALDH1A1 and CD133 (n=1).


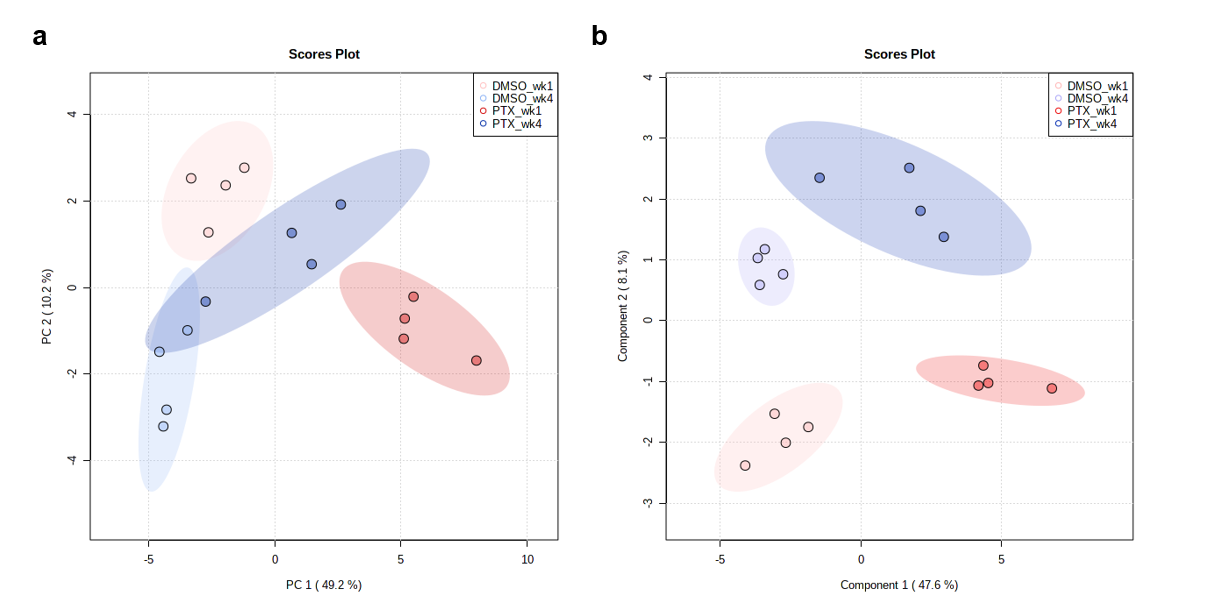


**Supplemental Figure 6. Supervised testing validates adequate sample separation.** Partial least squares discriminant analysis (PLSDA) plot of DMSO and PTX treated samples at 1 and 4 weeks; (n=4). Data was normalized to DMSO 1 week samples, log10 transformed, and scaled using the Pareto method. P-value < 0.05, FDR < 0.05 cut-offs were used.

**Supplemental Table 1. List of identified metabolites that were differentially abundant across 4 conditions.** One-way ANOVA; cut offs used p<0.05 and FDR>0.05; generated using MetaboAnalyst.

| **Metabolite** | **f.value** | **p.value** | **-LOG10(p)** | **FDR** |
| --- | --- | --- | --- | --- |
| Glutathione Reduced | 105.81 | 6.74E-09 | 8.1711 | 6.54E-07 |
| N-Acetylmethionine | 49.166 | 5.14E-07 | 6.2887 | 2.50E-05 |
| Psicose | 44.309 | 9.11E-07 | 6.0405 | 2.95E-05 |
| Citrate | 28.023 | 1.05E-05 | 4.9778 | 0.000236 |
| Isocitrate | 27.261 | 1.21E-05 | 4.9156 | 0.000236 |
| Phenylalanine | 23.441 | 2.63E-05 | 4.5798 | 0.000425 |
| Methyl Galactoside | 22.171 | 3.48E-05 | 4.4578 | 0.000483 |
| Beta-Alanine | 21.229 | 4.33E-05 | 4.3635 | 0.000525 |
| Thymine | 20.238 | 5.49E-05 | 4.2605 | 0.000592 |
| Taurine | 19.68 | 6.30E-05 | 4.2007 | 0.000611 |
| Nicotinamide | 18.909 | 7.66E-05 | 4.1157 | 0.000676 |
| Diiodotyrosine | 17.52 | 0.000111 | 3.9552 | 0.000784 |
| Glucose 1-Phosphate | 17.348 | 0.000116 | 3.9346 | 0.000784 |
| Dihydroorotate | 17.299 | 0.000118 | 3.9287 | 0.000784 |
| S-Adenosylmethionine | 17.197 | 0.000121 | 3.9164 | 0.000784 |
| Proline | 16.461 | 0.000149 | 3.8259 | 0.000869 |
| Fumarate | 16.394 | 0.000152 | 3.8174 | 0.000869 |
| Glucaric acid | 16.136 | 0.000164 | 3.7848 | 0.000885 |
| Ribitol | 15.606 | 0.000192 | 3.7165 | 0.000911 |
| 3-Hydroxymethylglutarate | 15.574 | 0.000194 | 3.7123 | 0.000911 |
| Inosine Monophosphate | 15.519 | 0.000197 | 3.7052 | 0.000911 |
| Lithocholate | 15.083 | 0.000225 | 3.6474 | 0.000993 |
| 4-hydroxyproline | 14.248 | 0.000293 | 3.5329 | 0.001236 |
| Carnosine | 13.47 | 0.000379 | 3.4217 | 0.001531 |
| Mannose | 13.348 | 0.000395 | 3.4038 | 0.001531 |
| Uridine Monophosphate | 13.179 | 0.000418 | 3.3788 | 0.00156 |
| Mannitol | 12.917 | 0.000458 | 3.3396 | 0.001644 |
| Benzoate | 12.518 | 0.000526 | 3.2787 | 0.001824 |
| Quinate | 11.833 | 0.000675 | 3.1708 | 0.002257 |
| AMP | 11.596 | 0.000737 | 3.1324 | 0.002384 |
| Tyrosine | 11.1 | 0.000891 | 3.0502 | 0.002743 |
| Elaidate | 11.059 | 0.000905 | 3.0434 | 0.002743 |
| N-Acetylaspartate | 10.89 | 0.000967 | 3.0146 | 0.002842 |
| Galactose | 10.673 | 0.001054 | 2.9774 | 0.003006 |
| Sebacate | 10.512 | 0.001124 | 2.9494 | 0.003077 |
| Deoxycarnitine | 10.472 | 0.001142 | 2.9423 | 0.003077 |
| Creatine | 10.218 | 0.001266 | 2.8974 | 0.00332 |
| Methionine | 9.196 | 0.001957 | 2.7085 | 0.004781 |
| Arginine | 9.1923 | 0.00196 | 2.7078 | 0.004781 |
| Serine | 9.1356 | 0.00201 | 2.6969 | 0.004781 |
| Guanosine | 9.123 | 0.002021 | 2.6945 | 0.004781 |
| Glycine | 8.7501 | 0.00239 | 2.6216 | 0.005481 |
| Uridine | 8.714 | 0.00243 | 2.6145 | 0.005481 |
| L-Carnitine | 8.6266 | 0.002529 | 2.597 | 0.005575 |
| Glutamate | 8.5445 | 0.002627 | 2.5806 | 0.005662 |
| Palmitoleate | 8.2527 | 0.003011 | 2.5212 | 0.00635 |
| Cystine | 7.9704 | 0.003448 | 2.4625 | 0.007115 |
| O-Phosphoethanolamine | 7.8608 | 0.003636 | 2.4393 | 0.007349 |
| 5-Hydroxyindoleacetate | 7.6128 | 0.00411 | 2.3862 | 0.008071 |
| FAD | 7.5884 | 0.00416 | 2.3809 | 0.008071 |
| Threonine | 7.4996 | 0.00435 | 2.3616 | 0.008273 |
| Adenosine | 7.4268 | 0.004512 | 2.3456 | 0.008417 |
| Galactosamine | 7.0147 | 0.005579 | 2.2534 | 0.010211 |
| Sucrose | 6.7799 | 0.006318 | 2.1994 | 0.011348 |
| Laurate | 6.6924 | 0.006622 | 2.179 | 0.011678 |
| Pyridoxal | 6.5509 | 0.00715 | 2.1457 | 0.012384 |
| Glutamine | 6.4519 | 0.007548 | 2.1221 | 0.012846 |
| Malate | 6.3408 | 0.008027 | 2.0954 | 0.013425 |
| Aspartate | 6.2191 | 0.008592 | 2.0659 | 0.014126 |
| Hypoxanthine | 6.0956 | 0.009214 | 2.0356 | 0.014895 |
| Inosine | 5.9846 | 0.009817 | 2.008 | 0.015611 |
| Oxalic Acid | 5.9424 | 0.010059 | 1.9975 | 0.015737 |
| Glycerol 3-Phosphate | 5.6582 | 0.011877 | 1.9253 | 0.018286 |
| N-Acetylglutamate | 5.51 | 0.012975 | 1.8869 | 0.019665 |
| Asparagine | 4.973 | 0.018074 | 1.7429 | 0.026972 |
| Pantothenate | 4.9297 | 0.018577 | 1.731 | 0.027302 |
| Sorbose | 4.5578 | 0.023649 | 1.6262 | 0.034238 |
| Cysteine | 4.5339 | 0.024025 | 1.6193 | 0.034272 |
| ADP | 4.3571 | 0.027045 | 1.5679 | 0.038019 |
| beta-Glycerophosphoric acid | 4.2685 | 0.028723 | 1.5418 | 0.039802 |
| Cytosine | 4.2426 | 0.029235 | 1.5341 | 0.039941 |
| Creatinine | 4.197 | 0.030164 | 1.5205 | 0.040637 |
| 4-Guanidinobutanoate | 4.0748 | 0.032827 | 1.4838 | 0.043619 |
| Methylthioadenosine | 3.9212 | 0.036567 | 1.4369 | 0.047932 |


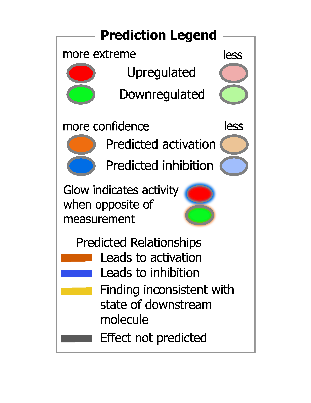

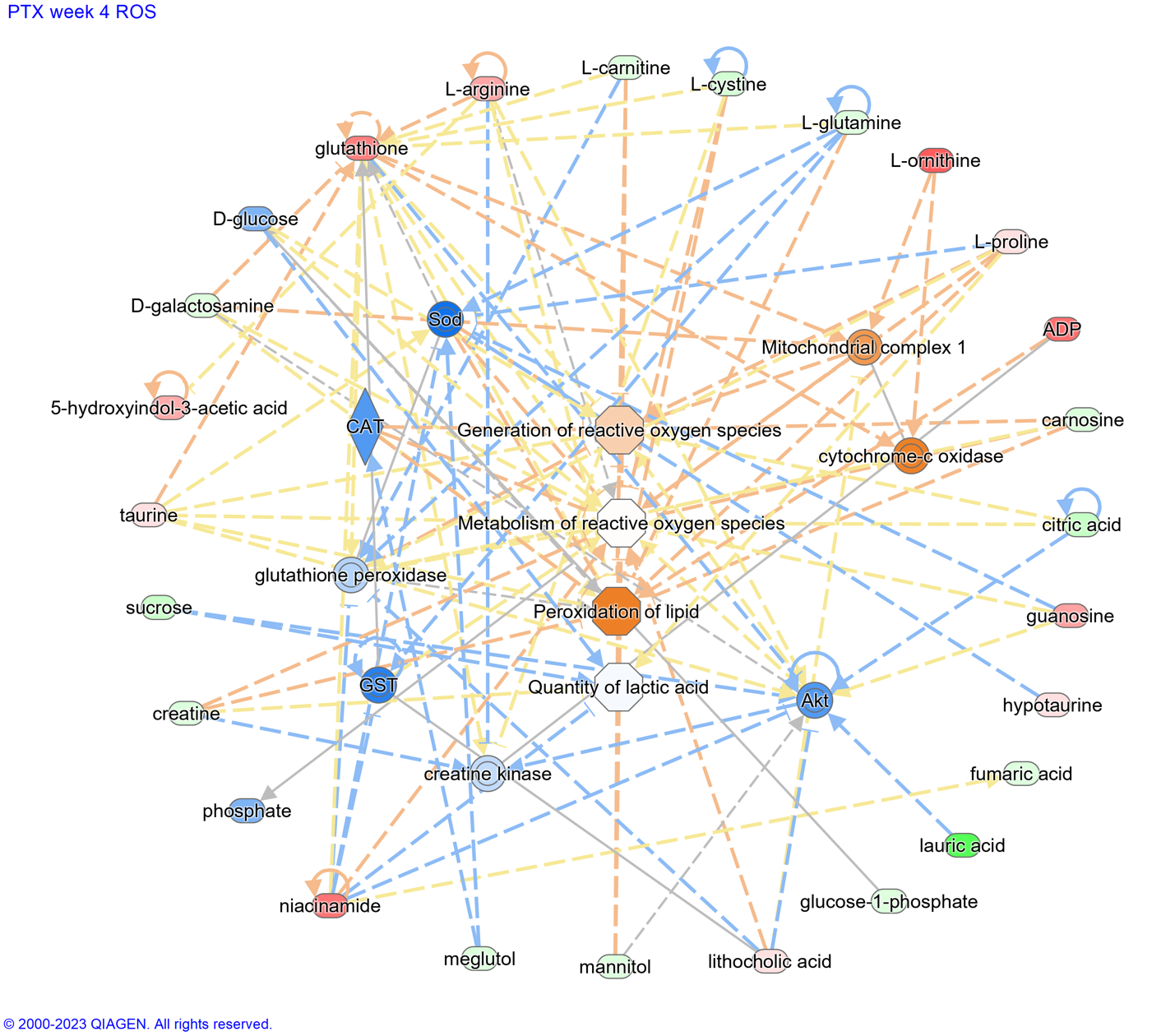


**Supplemental Figure 7. IPA generated oxidative stress network:** DMSO week 1

Data was analyzed using QIAGEN IPA (QIAGEN Inc., https://digitalinsights.qiagen.com/IPA).


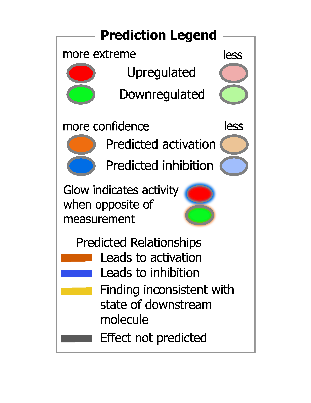

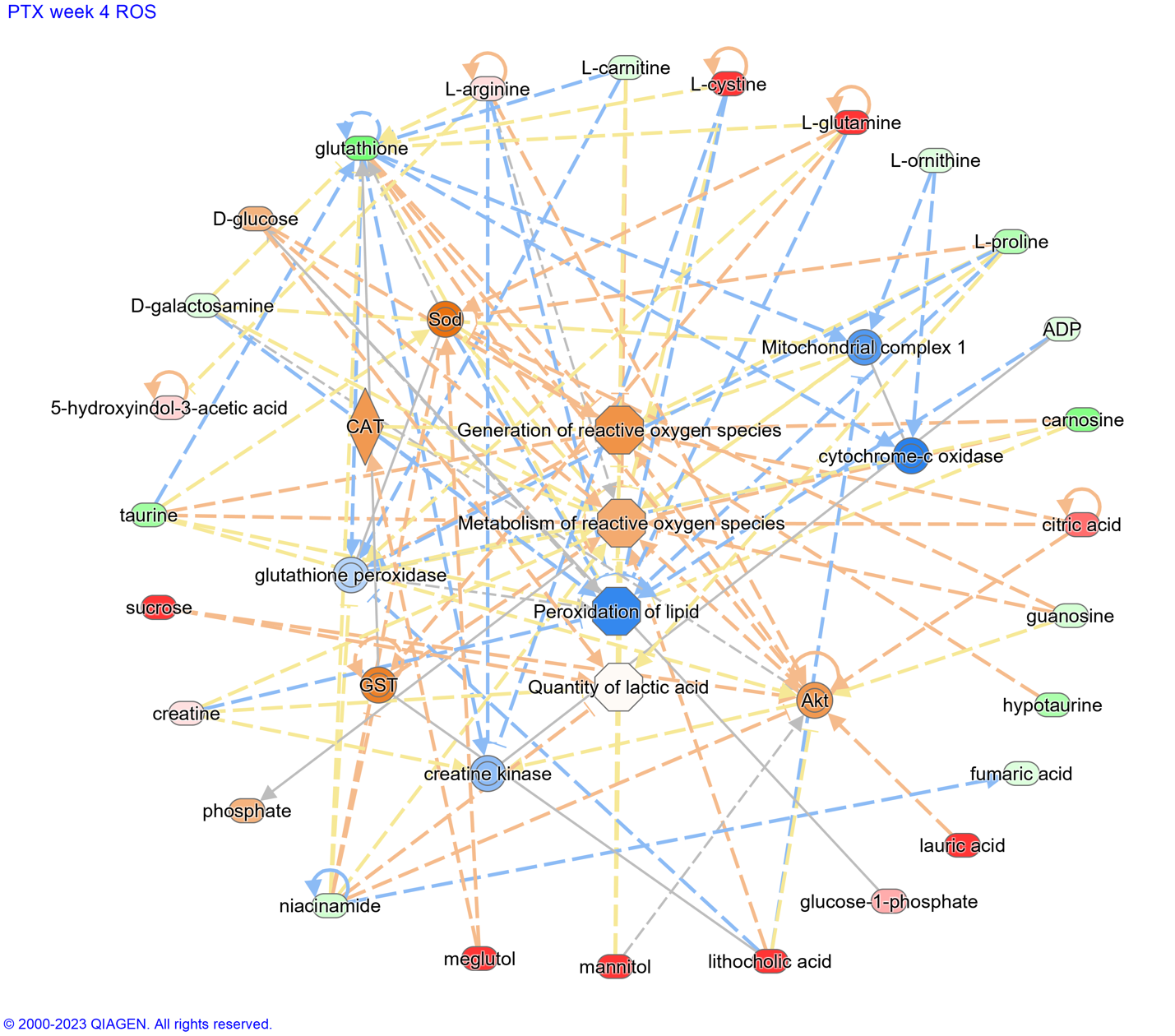


**Supplemental Figure 8. IPA generated oxidative stress network:** PTX week 1

Data was analyzed using QIAGEN IPA (QIAGEN Inc., https://digitalinsights.qiagen.com/IPA).


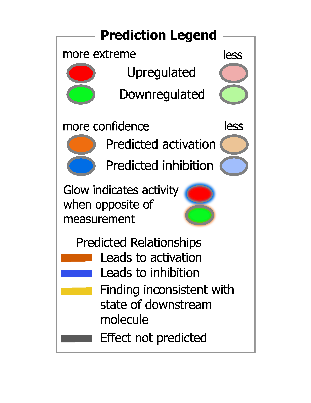

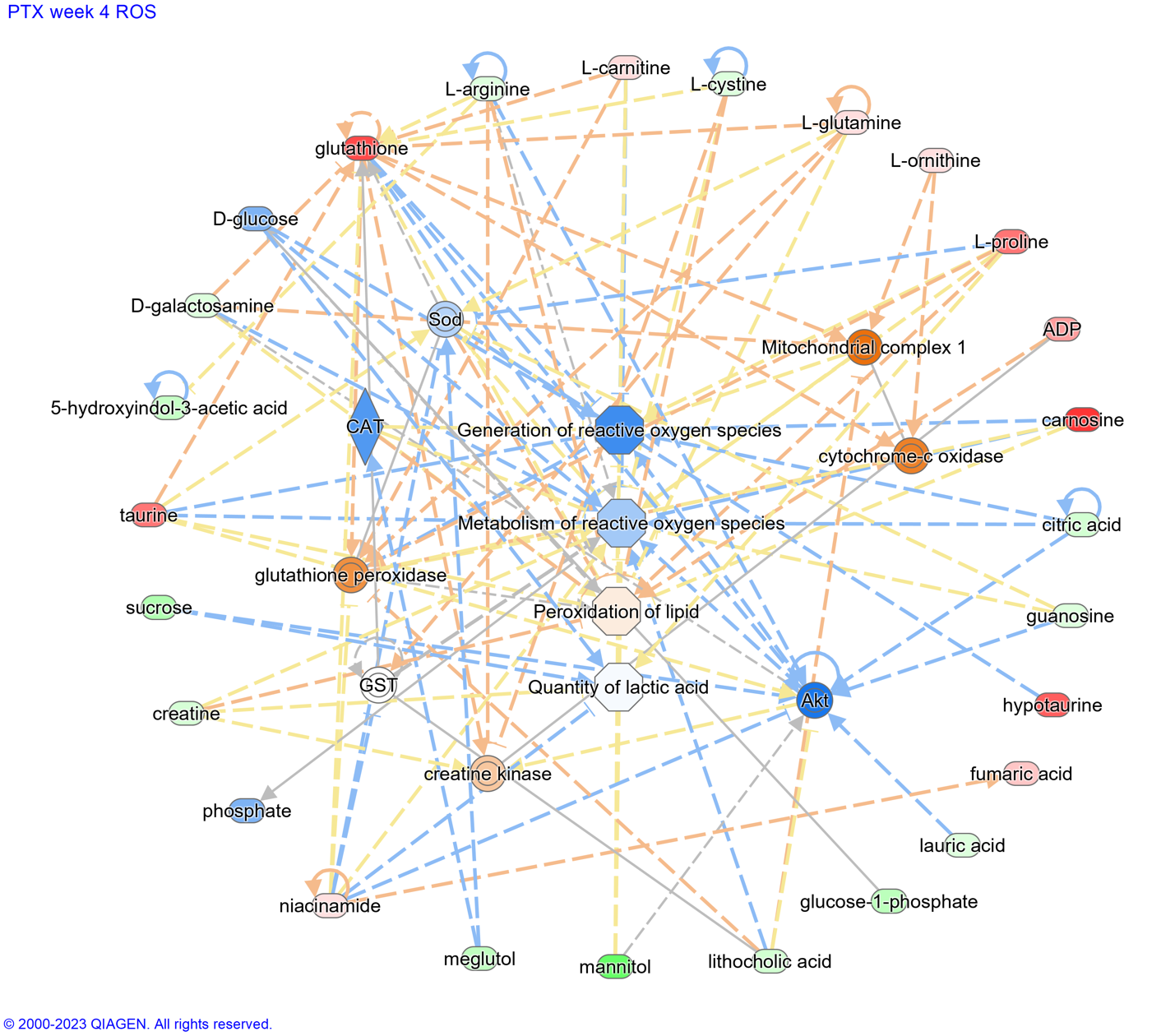


**Supplemental Figure 9. IPA generated oxidative stress network:** DMSO week 4

Data was analyzed using QIAGEN IPA (QIAGEN Inc., https://digitalinsights.qiagen.com/IPA).


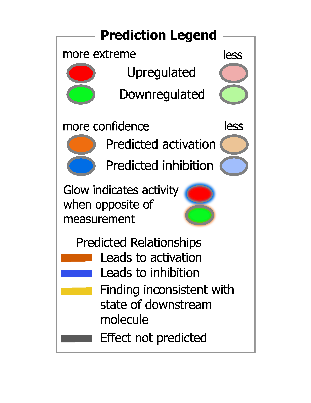

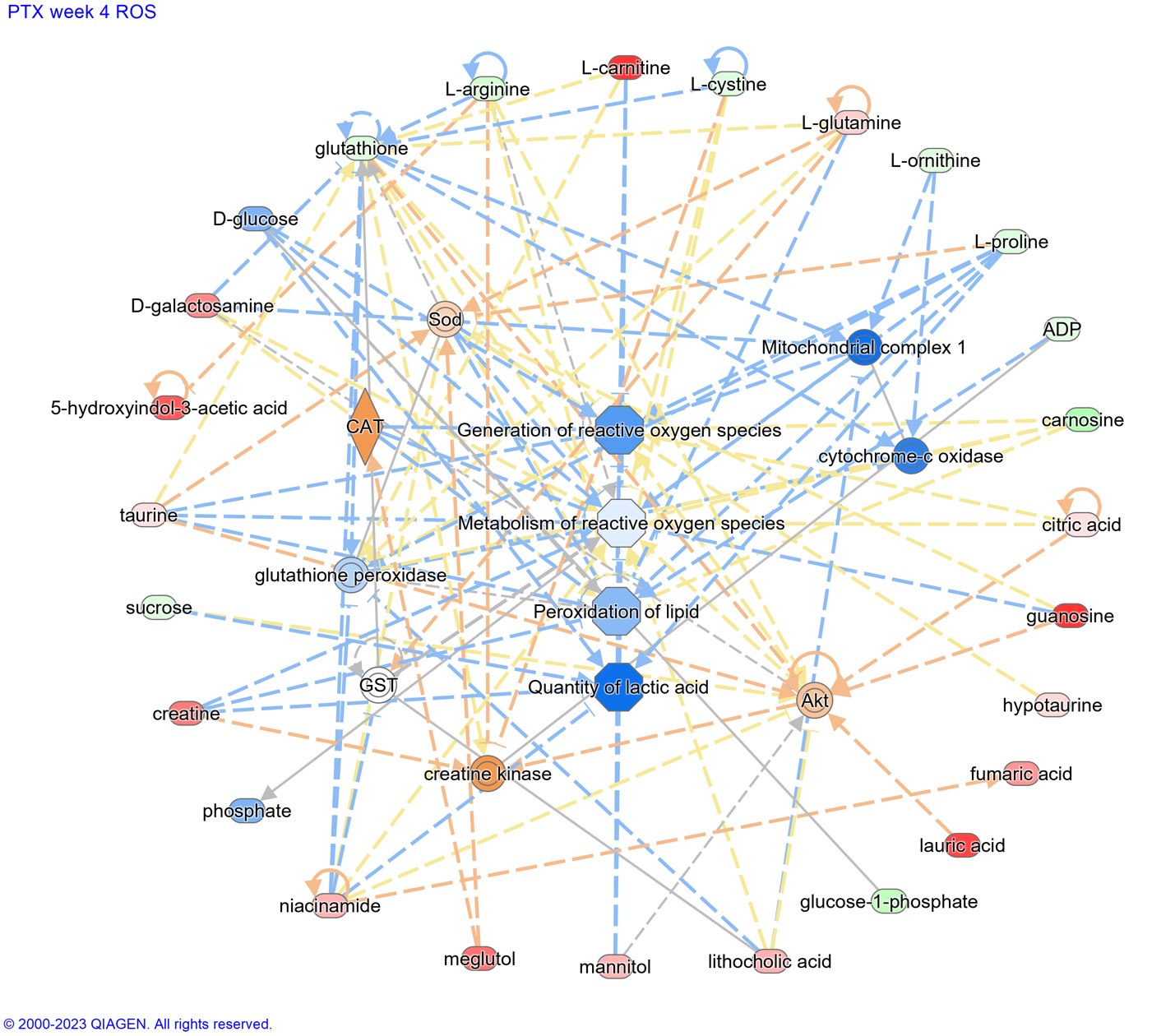


**Supplemental Figure 10. IPA generated oxidative stress network:** PTX week 4

Data was analyzed using QIAGEN IPA (QIAGEN Inc., https://digitalinsights.qiagen.com/IPA).

**Supplemental Table 2.**  Normalized abundancies of metabolites comprising the network generated using the interaction network analysis feature of QIAGEN IPA (QIAGEN Inc., https://digitalinsights.qiagen.com/IPA).

| **Metabolite** | **DMSO week 1** | **25-SING week 1** | **DMSO week 4** | **25-SING week 4** |
| --- | --- | --- | --- | --- |
| glutathione | 0.421721503 | -1.32276335 | 0.701729193 | 0.19931265 |
| D-galactosamine | -0.779124288 | 0.796395233 | -0.567387345 | 0.550116398 |
| 5-hydroxyindol-3-acetic acid (5-Hydroxyindoleacetate) | 0.179763303 | 0.276156373 | -0.495976023 | 0.04005635 |
| taurine | 0.224482658 | -1.041602898 | 0.559647813 | 0.257472423 |
| sucrose | -0.406288425 | 0.82090945 | -0.63903155 | 0.224410525 |
| creatine | -0.282235923 | 0.311387438 | -0.21646573 | 0.187314215 |
| niacinimide (nicotinamide) | 0.531752163 | -0.441390127 | -0.091080662 | 0.000718626 |
| meglutol (3-Hydroxymethylglutarate) | -0.379724958 | 0.950756995 | -0.643620593 | 0.072588548 |
| mannitol | -0.352604123 | 0.994741075 | -1.02564182 | 0.383504868 |
| lithocholic acid | -0.121299333 | 0.9070575 | -0.846403388 | 0.06064522 |
| glucose-1-phosphate | 0.13887291 | 0.588607758 | -0.222790173 | -0.504690493 |
| lauric acid | -0.520655905 | 0.780786233 | -0.431345513 | 0.171215185 |
| fumaric acid | -0.112815778 | -0.402378783 | 0.195033143 | 0.320161418 |
| hypotaurine | 0.271287325 | -0.738604375 | 0.085743825 | 0.381573225 |
| guanosine | 0.02846361 | -0.527247363 | 0.05672313 | 0.442060618 |
| citric acid | -0.39278342 | 0.823337401 | -0.463736024 | 0.033182043 |
| carnosine | 0.32284419 | -1.266181294 | 0.98840968 | -0.045072576 |
| ADP | 0.45940129 | -0.475447108 | 0.148129078 | -0.13208326 |
| L-proline | 0.225018515 | -0.72380131 | 0.377771938 | 0.121010858 |
| L-ornithine | 0.432491223 | -0.313063555 | -0.019510428 | -0.09991724 |
| L-glutamine | -0.510144685 | 0.625161078 | -0.117735463 | 0.002719067 |
| L-cystine | -0.585765558 | 0.96443024 | -0.104286025 | -0.27437866 |
| L-carnitine | -0.308453545 | -0.226297893 | 0.06257003 | 0.47218141 |
| L-arginine | 0.269235343 | 0.237754576 | -0.062031452 | -0.444958466 |

**Supplemental Table 3.**  Activation z-scores of predicted upstream regulators visualized in Figure 5C. Data was analyzed using QIAGEN IPA (QIAGEN Inc., https://digitalinsights.qiagen.com/IPA).

| **Upstream Regulator** | **DMSO week 1** | **PTX week 1** | **DMSO week 4** | **PTX week 4** |
| --- | --- | --- | --- | --- |
| OGT | -0.905 | 3.317 | -2.714 | 2.111 |
| GATA4 | 1.633 | -2.449 | 2.449 | -1.633 |
| HPD | -1 | 2 | -2 | 1 |
